# IFT88/Kindlin-2 Signaling Prevents Mechanical Overloading-Induced PANoptosis of Nucleus Pulposus Cells by Activating FOXP1 SUMOylation

**DOI:** 10.7150/ijbs.132842

**Published:** 2026-04-16

**Authors:** Kanglu Li, Mingjue Chen, Chao Chen, Yihan Yu, Sheng Liu, Zengwu Shao, Xianyi Cai, Guozhi Xiao, Sheng Chen

**Affiliations:** 1 Department of Orthopaedics, Union Hospital, Tongji Medical College, Huazhong University of Science and Technology, Wuhan 430022, China.; 2 Department of Biochemistry, Homeostatic Medicine Institute, School of Medicine, Shenzhen Key Laboratory of Cell Microenvironment, Guangdong Provincial Key Laboratory of Cell Microenvironment and Disease Research, Southern University of Science and Technology, Shenzhen 518055, China.; 3 Department of Orthopaedics, The Second Qilu Hospital, Cheeloo College of Medicine, Shandong University, Jinan 250012, China.

**Keywords:** intervertebral disc, nucleus pulposus, PANoptosis, Kindlin-2, IFT88, FOXP1

## Abstract

Intervertebral disc (IVD) degeneration (IDD) involves nucleus pulposus (NP) cell death, but its mechanisms are unclear. PANoptosis is a novel cell death pattern whose role in IDD remains elusive. This study investigates whether PANoptosis contributes to mechanical overloading-induced NP cell death and explores its underlying mechanisms. We evaluated PANoptosis in NP cells from human degenerated IVD tissues, aged mice, lumbar spine instability model, and an *in vitro* mechanical compression system. Results indicated that PANoptosis-related proteins were upregulated in NP cells from degenerated IVDs, as well as in those subjected to mechanical overloading both *in vivo* and *in vitro*. To confirm its functional role, we inhibited PANoptosis by interfering PANoptosis sensor Z-DNA-binding protein 1 (ZBP1). Results showed that ZBP1 inhibition partially reversed this upregulation, reduced NP cell death, and alleviated IDD. Mechanistically, we found Kindlin-2 loss promoted PANoptosis in NP cells by suppressing forkhead box P1 (FOXP1) SUMOylation and increasing ZBP1 promoter activity. Furthermore, mechanical overloading downregulated Kindlin-2 by impairing ciliary intraflagellar transport 88 (IFT88), thereby exacerbating PANoptosis and IDD. We show that ciliary IFT88 influences Kindlin-2, which protects NP cells from mechanical overloading—induced PANoptosis by enhancing FOXP1 SUMOylation. This pathway may offer a new therapeutic target for IDD.

## 1. Introduction

Low back pain (LBP) is one of the most prevalent musculoskeletal disorders and the leading global cause of years lived with disability^1^, ^2^. Intervertebral disc (IVD) degeneration (IDD) is recognized as one of the primary etiological factors of LBP^3^, ^4^. Due to limited understanding of the pathogenesis of IDD, current therapeutic strategies fail to achieve fundamental and long-term treatment outcomes. Therefore, in-depth investigation of the mechanisms underlying the initiation and progression of IDD is crucial for developing novel prevention and treatment strategies, holding significant scientific importance.

Mechanical stress constitutes the predominant microenvironment for IVD cells, with the nucleus pulposus (NP) serving as a critical structure maintaining disc biomechanical properties^5^. NP cell death induced by mechanical overloading represents a hallmark feature of IDD. Our previous studies, along with those from other research groups, have demonstrated that multiple regulated cell death modalities participate in NP cell death^6^-^8^. Among these, pyroptosis, apoptosis, and necroptosis currently represent the three most extensively studied and predominant forms of NP cell death. However, individual targeting of any single death modality has shown limited efficacy in reversing IDD progression. Recent studies from our team and others have revealed crosstalk among pyroptosis, apoptosis, and necroptosis^9^-^11^. It is now understood that key molecular effectors, such as caspases and RIPKs, operate within a highly interconnected network where activation of one pathway can potentiate or, conversely, inhibit another. This intricate interplay not only complicates the landscape of NP cell death but also explains the limited success of therapies designed to block any single RCD modality, as blocking one route may shunt cellular stress toward an alternative lethal pathway. These findings suggest the necessity to move beyond traditional single-death-paradigm research and re-examine the mechanisms of mechanical overloading-induced NP cell death. Identifying core regulatory nodes and key targets capable of coordinately modulating multiple cell death pathways represents the critical approach for effectively rescuing NP cell death and treating IDD.

PANoptosis, a novel regulated cell death modality first conceptualized in 2019 by inflammasome research pioneer Thirumala-Devi Kanneganti and colleagues, encompasses key regulatory molecules from pyroptosis, apoptosis, and necroptosis pathways^12^, ^13^. Under pathological microenvironmental stimuli, PANoptosis sensors like Z-DNA-binding protein 1 (ZBP1) initiate the assembly of the PANoptosome^14^. This molecular complex integrates components from three cell death pathways: receptor-interacting protein kinase (RIPK) 1/RIPK3, Fas-associated protein with death domain (FADD)/caspase-8 (CASP8), and NOD-like receptor family pyrin domain-containing 3 (NLRP3)/apoptosis-associated speck-like protein containing a CARD (ASC)/CASP1. It subsequently activates effector molecules, including mixed lineage kinase domain-like protein (MLKL), CASP3/CASP7/gasdermin E (GSDME), and GSDMD, to execute cell death^15^-^17^. Distinct from conventional single-pathway paradigms, PANoptosis provides a unified framework connecting pyroptosis, apoptosis, and necroptosis, making it a more efficient therapeutic target for comprehensive cell death inhibition. While significant breakthroughs have been achieved in PANoptosis research across various fields^18^, ^19^, its potential involvement in mechanical overloading-induced NP cell death during IDD remains unexplored, with the underlying molecular mechanisms yet to be elucidated.

Therefore, in the current study, through comprehensive molecular and histological analyses employing both gain- and loss-of-function strategies, combined with multiple IDD models, we establish that PANoptosis mediates mechanical overloading-induced NP cell death in IDD pathogenesis. Under mechanical overloading, ciliary intraflagellar transport 88 (IFT88) regulates Kindlin-2 to promote SUMOylation of forkhead box P1 (FOXP1) at lysine 636. This process suppresses PANoptosis in nucleus pulposus cells. Our findings may identify a novel therapeutic target for IDD.

## 2. Materials and Methods

### 2.1 Human NP samples

A total of 16 human NP samples were collected from patients undergoing nucleotomy for lumbar disc herniation (7 males, 9 females; age range: 33-73 years, Supplementary Table 1). To minimize potential confounding effects of systemic diseases and external physical trauma on NP cell death, subjects with a history of spinal injury, prior lumbar surgery, spinal infection, malignancy, or systemic comorbidities (including diabetes mellitus and severe osteoporosis) were excluded from this study. Degenerative status was assessed via MRI using the Pfirrmann grading system: specimens were categorized into mild IDD (Grades II, N=2; Grades III, N=6) and severe IDD (Grades IV, N=4; Grades V, N=4) groups^11^. This study was approved by the Ethics Committee of Tongji Medical College, Huazhong University of Science and Technology (Approval No. [2023] IEC (140)), with written informed consent obtained from all participants.

### 2.2 Animal studies

The generation of* Kindlin-2^fl/fl^; Aggrecan^CreERT2^* mice and *Aggrecan^CreERT2^* mice was conducted as previously reported^11^, ^20^. The *Ift88^fl/fl^* mice were purchased from Shanghai Model Organisms Center. To achieve conditional deletion of IFT88 in aggrecan-expressing cells, *Ift88^fl/fl^* mice were first crossed with *Aggrecan^CreERT2^* mice, resulting in the generation of *Ift88^fl/+^; Aggrecan^CreERT2^*mice. These mice were subsequently backcrossed with *Ift88^fl/fl^* mice to obtain *Ift88^fl/fl^; Aggrecan^CreERT2^* mice (Supplementary Figure 1). At two months of age, male* Kindlin-2^fl/fl^; Aggrecan^CreERT2^* and *Ift88^fl/fl^; Aggrecan^CreERT2^* mice were administered five consecutive daily intraperitoneal (i.p.) injections of tamoxifen (Sigma-Aldrich; T5648) at a dose of 100 mg/kg body weight. This treatment was aimed at inducing the deletion of Kindlin-2 and IFT88 expression, respectively, in aggrecan-positive cells. As controls, sex- and age-matched *Kindlin-2^fl/fl^; Aggrecan^CreERT2^* or *Ift88^fl/fl^; Aggrecan^CreERT2^* were treated with corn oil (Sigma-Aldrich; C8267) under identical conditions. All mice utilized in this study had been backcrossed with normal C57BL/6 mice for a minimum of ten generations to ensure genetic homogeneity. Primer information is provided in Supplementary Table 2. To minimize animal usage and maintain experimental consistency, only male mice were selected for all experiments. The mice were housed in groups under controlled environmental conditions, with a temperature range of 20-24°C and a 12-hour light/12-hour dark cycle. All experimental protocols involving animals were approved by the Institutional Animal Care and Use Committee (IACUC) of Huazhong University of Science and Technology (Approval No. [2023] IACUC (3995).

### 2.3 Lumbar spine instability IDD model

Briefly, anesthesia was induced using 2.5% avertin prior to surgical procedures. For the lumbar spine instability (LSI) IDD model, male C57BL/6 mice aged 3 months underwent surgical resection of the spinous processes at the lumbar 3rd to lumbar 5th (L3-L5) levels, along with the excision of the supraspinous and interspinous ligaments. In contrast, sham-operated mice served as controls and underwent only the detachment of the posterior paravertebral muscles from the L3-L5 vertebrae without any further disruption to the spinal column^21^. To investigate the impact of Kindlin-2 or IFT88 deletion on mice subjected to abnormal mechanical loading conditions, two-month-old *Kindlin-2^fl/fl^; Aggrecan^CreERT2^* or *Ift88^fl/fl^; Aggrecan^CreERT2^* mice were treated with either tamoxifen or corn oil via i,p. injection to induce gene deletion or serve as controls, respectively. One month following the gene modulation treatment, the LSI-IDD model was established in these mice using the aforementioned surgical approach. All experimental mice were euthanized one month post-surgery, and IVD samples were harvested.

### 2.4 Coccygeal IVD compression IDD model

A coccygeal IVD compression (CIC)-induced IDD model was established in 3-month-old Sprague Dawley rats as previously described^11^. Briefly, rats were anesthetized with isoflurane (RWD; R510-22-4), and carbon fiber rings were fixed to coccygeal vertebrae Co8 and Co9 using sterile 0.8-mm Kirschner wires. Axial compression (1.3 MPa) was applied via four 0.50-N/mm calibrated springs mounted on each rod. To investigate the role of ZBP1, rats received intradiscal injections (33-gauge needle, Hamilton, Switzerland) of either negative control (NC) or ZBP1 siRNA three times weekly for two weeks. Primer information is provided in Supplementary Table 2. Sham-operated controls were injected with PBS and fitted with the loading device but without compressive force. All animals were euthanized two weeks post-surgery for further analysis.

### 2.5 Cell culture and treatments

Human primary NP cells were isolated from surgically obtained NP tissues using established protocols^22^. Cells were expanded in DMEM/F-12 medium (Hyclone; SH30023.01) supplemented with 10% fetal bovine serum (FBS; Gibco, 10099-141) and 1% penicillin-streptomycin (Hyclone; SV30010) at 37°C in a 5% CO₂ humidified incubator. The medium was refreshed every three days, and cells were subcultured at a 1:2 ratio upon reaching 80% confluence. Second-passage NP cells were used for all experiments. HEK293T cells were maintained in DMEM (Corning; 10-013-CVR) containing 10% FBS and 1% penicillin-streptomycin under identical culture conditions, as described in previous study^23^. To simulate pathological mechanical overloading, NP cells with or without siRNA or plasmid transfection were subjected to 1.0 MPa compressive loading for 24 hours using a custom-designed compression device (Chinese Patent No. ZL 201120082425.3)^11^. NP cells were seeded in culture plates and placed within the apparatus under standard incubation conditions (37°C, 5% CO₂). For siRNA transfection, cells were transfected with NC, Kindlin-2 or ZBP1 siRNAs at a concentration of 50 pmol/10^5^ cell using Lipofectamine RNAiMAX Transfection Reagent (ThermoFisher Scientific; 13778150). Primer information is provided in Supplementary Table 2. For plasmid transfection, cells were transfected with NC, Kindlin-2, FOXP1-WT or FOXP1^K636R^ plasmid at a concentration of 5 μg/10^5^ cells using LipofectamineTM 3000 Transfection Reagent (Invitrogen; L3000015).

### 2.6 Histology and immunostaining assays

Human NP tissues, mouse and rat IVDs were fixed in 4% paraformaldehyde, processed through graded ethanol dehydration, and embedded in paraffin. Sections (5 μm) were prepared for histological analysis. Human NP sections were stained with hematoxylin and eosin (H&E), while mouse and rat IVD sections were stained with safranin O/fast green (SO/FG), and histological grading was performed according to established scoring systems^8^. For immunofluorescence (IF) analysis, tissue sections were deparaffinized in xylene and rehydrated through a graded ethanol series. Antigen retrieval was performed using citrate buffer. After blocking with QuickBlock™ Blocking Buffer (Beyotime) containing 0.1% Triton X-100, sections were incubated with primary antibodies at 4°C overnight, followed by appropriate fluorescent secondary antibodies. Immunofluorescence images were acquired using a Nikon A1R confocal microscope and analyzed with ImageJ software. The cell counts were obtained through manual counting by two independent, blinded observers. Cilium length and prevalence were quantified from maximum-intensity projections of confocal z-stacks acquired using a 100× oil immersion objective. Ciliary length was measured using ImageJ software, while cilium prevalence was determined from the same projections. Antibody information is provided in Supplementary Table 3.

### 2.7 Propidium iodide staining

Propidium iodide (PI) staining was performed to detect cell death as described previously^24^, ^25^. For *in vitro* experiments, NP cells were washed with PBS and treated with 10 μg/mL PI (Beyotime, ST512) in DMEM/F-12 medium and incubated for 20 min at 37°C (5% CO₂), protected from light. For tissue analysis, human NP specimens and rodent IVDs were treated with 100 μg/mL PI in DMEM/F-12 medium incubated for 3 hours at 37°C (5% CO₂). Following PBS washes, tissues were processed for cryosections. Fluorescence imaging was performed using a Nikon A1R confocal microscope, with quantitative analysis conducted using ImageJ software.

### 2.8 Western blotting analyses

Protein extracts from NP or HEK293T cells were analyzed by western blotting. Following centrifugation (13000 rpm, 10 minutes, 4°C), protein concentrations were determined using the DC protein assay. Equal amounts of protein were separated by SDS-PAGE and transferred to polyvinylidene fluoride membranes (Bio-Rad Laboratories). After blocking, membranes were incubated with primary antibodies overnight at 4°C, followed by three TBST washes and 1 h incubation with HRP-conjugated secondary antibodies at room temperature. Protein bands were visualized using enhanced chemiluminescence detection. All antibodies used are listed in Supplementary Table 3.

### 2.9 Co-immunoprecipitation and SUMOylation assay

Briefly, cells were lysed in IP lysis buffer supplemented with protease inhibitor cocktail for 10 min at 4°C. The lysate was centrifuged at 13000 rpm for 10 min at 4°C to remove debris, and the supernatant was incubated with an anti-FOXP1 primary antibody overnight at 4°C with gentle rotation. Subsequently, Protein A/G Magnetic Beads were added to the lysate-antibody mixture and incubated for 1 h at room temperature. The bead-bound antigen-antibody complexes were isolated using a DynaMag-2 Magnet (Thermo Fisher Scientific) and washed three times with cold IP buffer. The immunoprecipitated complexes were then resuspended in 1× loading buffer, denatured at 95°C for 5 min, and subjected to SDS-PAGE followed by Western blotting. Membranes were probed with antibodies against SUMO-1 and FOXP1 to detect SUMOylated FOXP1. All antibodies used in this study are listed in Supplementary Table 3.

### 2.10 Dual-luciferase reporter assay

To investigate the regulatory role of FOXP1 on ZBP1 promoter activity, a 200-bp DNA fragment encompassing the predicted FOXP1 binding site (including both upstream and downstream flanking sequences) was cloned into the pGL3-Basic-Luc reporter vector. HEK293T cells were co-transfected using Lipofectamine 3000 Transfection Reagent (Invitrogen; L3000015) with the following plasmids: (1) the constructed ZBP1 promoter reporter (pGL3-ZBP1-Luc), (2) either wild-type FOXP1 or K636R mutant expression vectors, and (3) the Renilla luciferase control plasmid pRL-TK for normalization. After 48 hours post-transfection, dual-luciferase activity was measured using the Dual-Luciferase Reporter Assay System (Beyotime, RG027) according to the manufacturer's protocol. Firefly luciferase activity was normalized to Renilla luciferase activity for each sample, and relative promoter activity was expressed as the ratio of Firefly to Renilla luminescence units.

### 2.11 Statistical analysis

Data were expressed as mean ± standard deviation (SD) from a minimum of three independent experiments. The normality of data distribution was assessed using the Shapiro-Wilk (S-W) test. All the data conform to the normal distribution. Intergroup differences were analyzed using unpaired two-tailed Student's t-tests (two-group comparisons) or one-way ANOVA with Tukey's post hoc test (multi-group comparisons). In the present study, the sample size "n" consistently designates the number of independent biological replicates. Specifically, for *in vivo* animal experiments and human tissue analyses, n indicates the number of individual animals or distinct human donors comprising each experimental group, whereas for *in vitro* cell culture studies, n represents independent experiments conducted on separate occasions or utilizing cells isolated from different primary donors (with a minimum of three biological replicates performed). When technical replicates were generated within a single experiment, their values were averaged prior to statistical analysis to constitute a single biological replicate. All analyses were performed using GraphPad Prism 6 (GraphPad Software, Inc.), with statistical significance set at *p* < 0.05.

## 3. Results

### 3.1 PANoptosis-related proteins are highly expressed in NP cells in severe IDD patients and aged mice

**Figure 1:**
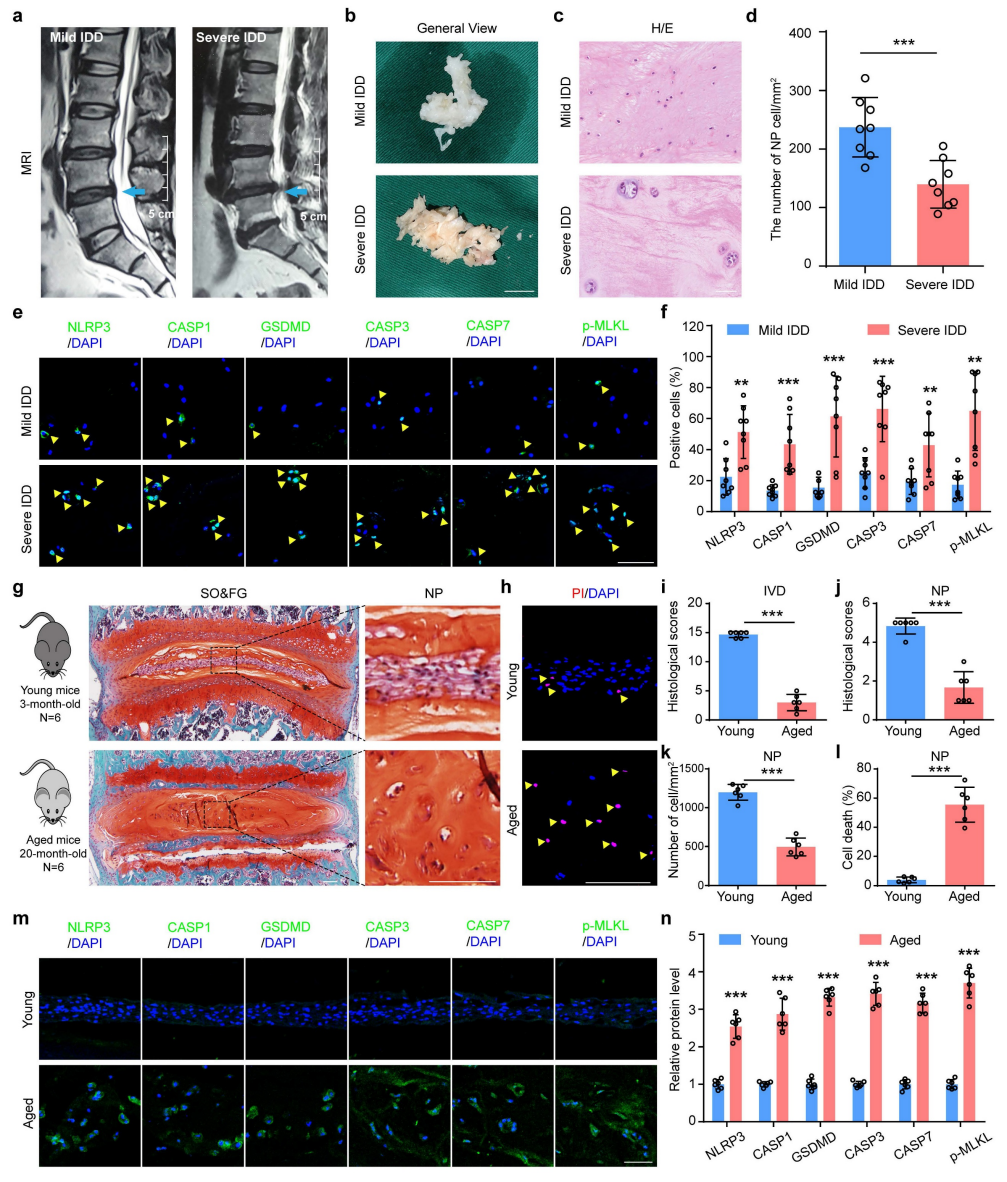
**PANoptosis of NP cells drastically increases in severe IDD patients and aged mice. (a)** Representative magnetic resonance imaging. Scale bar, 5 cm. **(b)** General views of human degenerated NP tissues. Scale bar, 1 cm. **(c)** Hematoxylin and eosin (H/E) staining of human NP samples. Scale bar, 50 μm. **(d)** The number of NP cells in human degenerated NP tissues (cells/mm^2^, n=8).** (e, f)** Immunofluorescent (IF) staining of NLRP3, CASP1, GSDMD, CASP3, CASP7, and p-MLKL in human NP samples (n=8). Scale bar, 50 μm. **(g, i, j)** Safranin O and Fast Green (SO&FG) staining and histological scores of IVD and NP in young mice (3-month-old) and aged mice (20-month-old). (n=6). Scale bar, 100 μm. **(k)** The number of NP cells in young and aged mice (cells/mm^2^, n=6). **(h, l)** Propidium iodide (PI) staining and cell death rate of NP in young and aged mice (n=6). Scale bar, 100 μm. **(m, n)** IF staining of NLRP3, CASP1, GSDMD, CASP3, CASP7, and p-MLKL in young and aged mice (n=6). Scale bar, 50 μm. Results are expressed as mean ± standard deviation (s.d.). **P < 0.01, ***P < 0.001.

To explore the potential role of PANoptosis in IDD, we collected 16 degenerative NP samples from IDD patients and analyzed the expression of key PANoptosis-related proteins, including NLRP3, CASP1, GSDMD, CASP3, CASP7, and p-MLKL. Prior to molecular analysis, the degenerative degree of the NP specimens was assessed and confirmed using the Pfirrmann grading system based on preoperative magnetic resonance imaging (MRI) (Figure 1a, b). Hematoxylin and eosin (H/E) staining revealed that the number of NP cells was significantly decreased in severe IDD group compared to that from mild IDD group (Figure 1c, d). Immunofluorescent (IF) staining showed that the protein expression levels of NLRP3, CASP1, GSDMD, CASP3, CASP7, and p-MLKL were drastically increased in human NP tissues from severe versus mild IDD samples (Figure 1e, f). Furthermore, results from safranin O and fast green (SO&FG) staining demonstrated that the histological scores of IVDs and NP tissues in aged mice (20-month-old) were significantly lower than those in young mice (3-month-old) (Figure 1g, i and j). A significant decrease in the number of NP cells in aged mice was also observed (Figure 1k). Aged mice were employed to validate pathological states inherent to natural senescence, thereby establishing clinical relevance through direct parallels with severe human IDD specimens. In addition, propidium iodide (PI) staining revealed that the cell death rate in NP tissues was obviously higher from aged mice than that in young mice (Figure 1h, l). Consistent with findings in human degenerated NP tissues, IF staining demonstrated a significant upregulation of PANoptosis-related proteins, including NLRP3, CASP1, GSDMD, CASP3, CASP7, and p-MLKL, in aged mice compared to young controls (Figure 1m, n).

### 3.2 Mechanical overloading promotes PANoptosis-related protein expressions and cell death in NP cells

**Figure 2:**
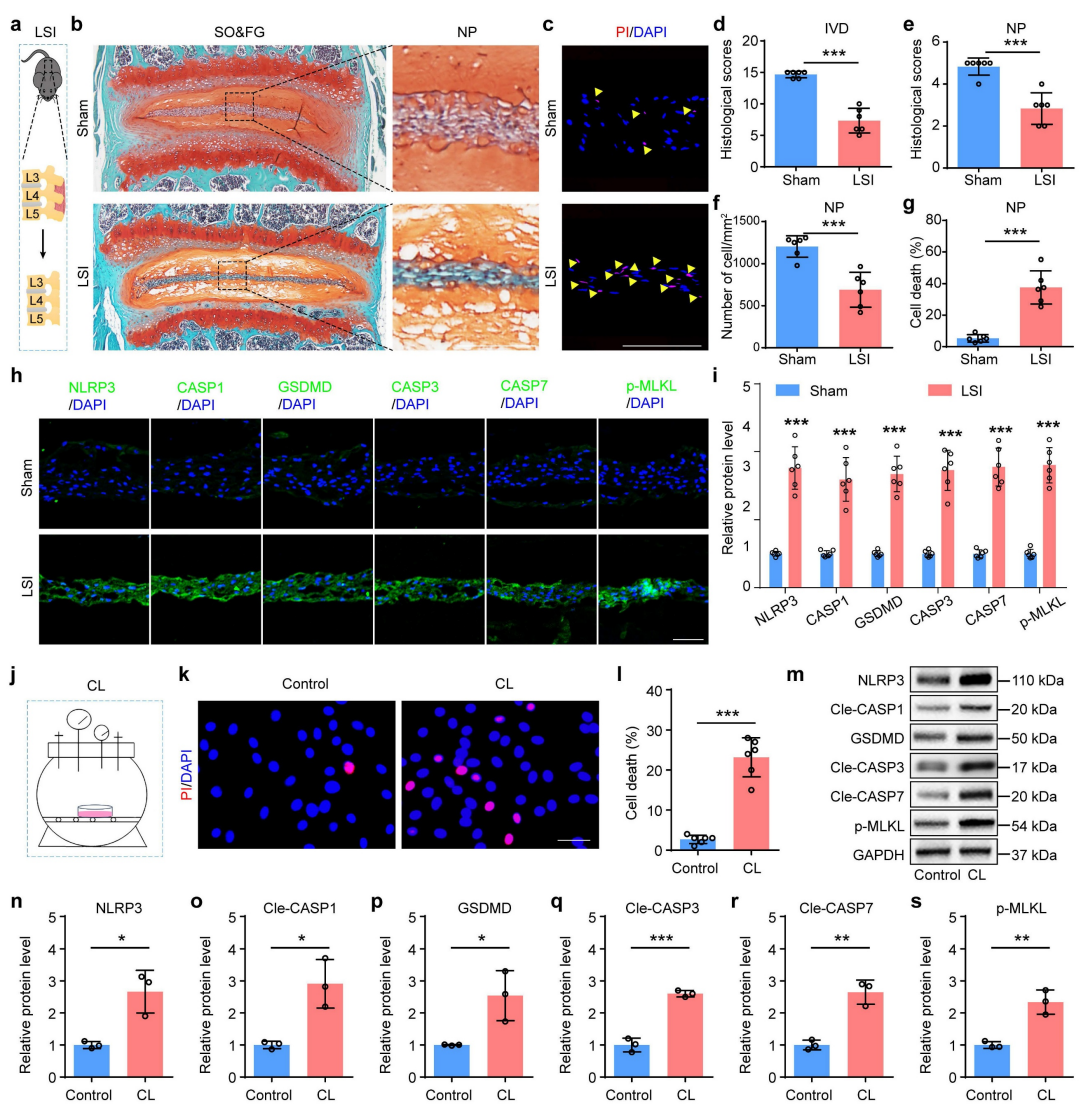
**Mechanical overloading promotes PANoptosis of NP cells and IDD. (a)** Lumbar spine instability (LSI) model. **(b, d, e)** SO&FG staining and histological scores of IVD and NP in sham and LSI mice (n=6). Scale bar, 100 μm. **(f)** The number of NP cells in sham and LSI mice (cells/mm^2^, n=6). **(c, g)** PI staining and cell death rate of NP in sham and LSI mice (n=6). Scale bar, 100 μm. **(h, i)** IF staining of NLRP3, CASP1, GSDMD, CASP3, CASP7, and p-MLKL in sham and LSI mice (n=6). Scale bar, 50 μm.** (j)** Compression loading (CL) apparatus.** (k, l)** Propidium iodide (PI) staining and cell death rate of NP in control and CL group (n=6). Scale bar, 50 μm.** (m-s)** Western blotting analyses of NLRP3, Cle-CASP1, GSDMD, Cle-CASP3, Cle-CASP7, and p-MLKL of NP in control and CL group (n=3). Results are expressed as mean ± standard deviation (s.d.). *P < 0.05, **P < 0.01, ***P < 0.001.

To further validate the involvement of PANoptosis in NP cell death under abnormal mechanical stress condition, we established lumbar spine instability (LSI) mice model to simulate mechanical overloading applied to IVDs *in vivo* (Figure 2a). Given that the lumbar spine represents the predominant anatomical site for human IDD, the LSI model optimally recapitulates the chronic, aberrant biomechanical loading characteristic of this region *in vivo*. SO&FG staining revealed significant reduction in histological scores of IVDs and NP tissues in LSI group relative to the sham group (Figure 2b, d and e). And a marked decrease in NP cell number was observed in LSI group (Figure 2f). PI staining confirmed a substantially higher rate of cell death in NP tissues from LSI mice than that in sham controls (Figure 2c, g). Moreover, IF staining showed significantly increased expressions of PANoptosis-related proteins, including NLRP3, CASP1, GSDMD, CASP3, CASP7, and p-MLKL, in LSI group compared to sham group (Figure 2h, i). Additionally, to mimic mechanical overloading conditions *in vitro*, we employed a customized compression apparatus (Figure 2j). We found that abnormal compression loading (CL) significantly promoted cell death (Figure 2k, l) and upregulated the expression levels of PANoptosis-related proteins (Figure 2m-s).

### 3.3 Inhibition of PANoptosis alleviates mechanical overloading-induced NP cell death and IDD

**Figure 3:**
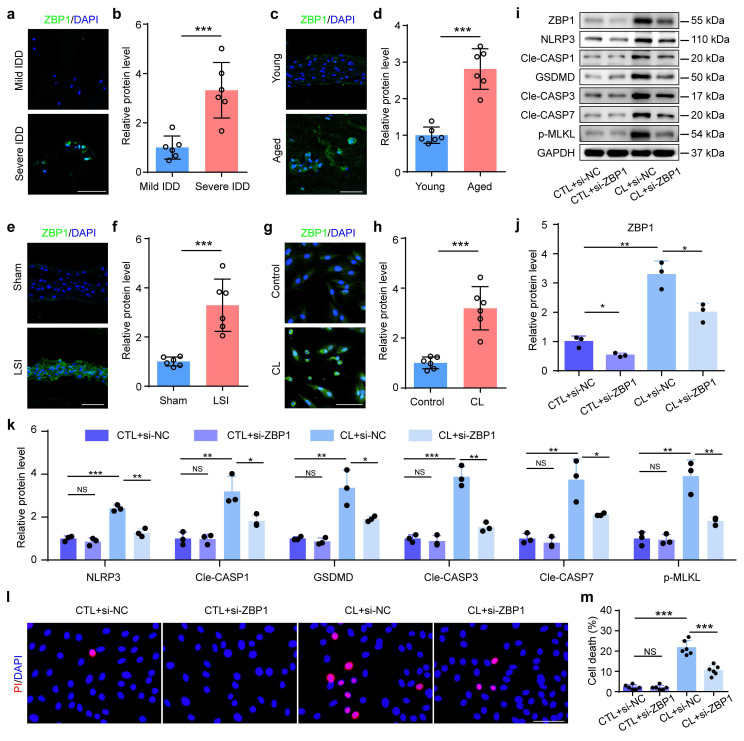
**Inhibition of ZBP1 alleviates mechanical overloading-induced PANoptosis in NP cells. (a, b)** IF staining of ZBP1 in human NP samples (n=8). Scale bar, 50 μm. **(c, d)** IF staining of ZBP1 in young and aged mice (n=6). Scale bar, 50 μm. **(e, f)** IF staining of ZBP1 in sham and LSI mice (n=6). Scale bar, 50 μm. **(g, h)** IF staining of ZBP1 in control and compression loading (CL) group (n=6). Scale bar, 50 μm.** (i-k)** Western blotting analyses of ZBP1, NLRP3, Cle-CASP1, GSDMD, Cle-CASP3, Cle-CASP7, and p-MLKL of NP cells, which were transfected with negative control siRNA (si-NC) or ZBP1 siRNA (si-ZBP1), and then treated with or without CL treatment (n=3). **(l, m)** Propidium iodide (PI) staining and cell death rate of NP cell, which were treated as in (i) (n=6). Scale bar, 50 μm. Results are expressed as mean ± standard deviation (s.d.). NS, no statistical significance, *P < 0.05, **P < 0.01, ***P < 0.001.

**Figure 4:**
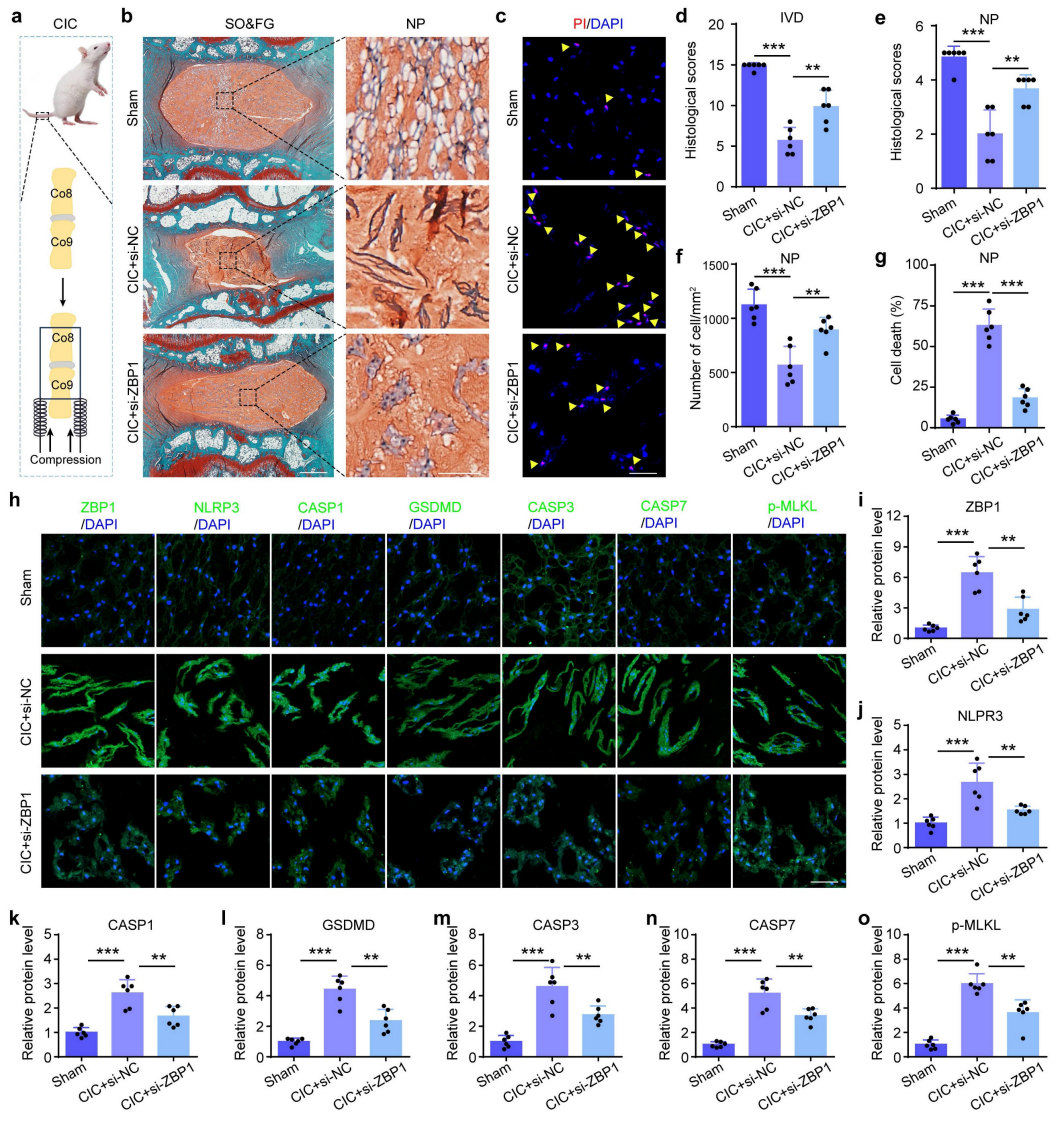
**Inhibition of ZBP1 alleviates mechanical overloading-induced NP cell PANoptosis and IDD in rat. (a)** Coccygeal IVD compression (CIC) model. **(b, d, e)** SO&FG staining and histological scores of IVD and NP in sham and CIC rats, which were treated with si-NC or si-ZBP1 (n=6). Scale bar, 500 or 100 μm. **(f)** The number of NP cells in rats treated as in (b) (cells/mm^2^, n=6). **(c, g)** PI staining and cell death rate of NP in rats treated as in (b) (n=6). Scale bar, 50 μm. **(h-o)** IF staining of ZBP1, NLRP3, CASP1, GSDMD, CASP3, CASP7, and p-MLKL in rats treated as in (b) (n=6). Scale bar, 50 μm. Results are expressed as mean ± standard deviation (s.d.). **P < 0.01, ***P < 0.001.

ZBP1 is a sensor protein and plays an essential role in the initiation of the PANoptosis. Results from IF staining showed that compared to control groups, the expression of ZBP1 was increased in NP cells from severe IDD patients, aged mice, LSI group, and CL group, respectively (Figure 3a-h). To further validate the functional significance of PANoptosis in NP cell death and IDD, we inhibited this process by using ZBP1 siRNA. Results from western blotting revealed that si-ZBP1 partially reversed the upregulation of the expressions of PANoptosis-related proteins caused by CL in NP cells, including NLRP3, Cle-CASP1, GSDMD, Cle-CASP3, Cle-CASP7, and p-MLKL (Figure 3i-k). PI staining demonstrated that si-ZBP1 alleviated CL-induced NP cell death (Figure 3l, m). In addition, we inhibited ZBP1 expression using si-ZBP1 in a coccygeal IVD compression (CIC) model, which simulated mechanical overloading applied to IVDs *in vivo* (Figure 4a). This model permits the application of precise, quantifiable mechanical stress. The histological evaluation via SO&FG staining demonstrated notably lower scores in the IVDs and NP tissues of the CIC group than those in the sham group (Figure 4b, d and e). Concurrently, a significant decline in NP cell number was detected in CIC group (Figure 4f). PI staining further corroborated an elevated rate of cell death in NP tissues from CIC mice compared to sham controls (Figure 4c, g). IF staining revealed upregulated expression levels of ZBP1, NLRP3, CASP1, GSDMD, CASP3, CASP7, and p-MLKL in CIC group relative to sham group (Figure 4h-o). Notably, these pathological alterations were effectively attenuated by si-ZBP1 intervention (Figure 4b-o). Thus, above results indicate that PANoptosis plays an important role in the development and progression of IDD, and mechanical overloading can promote ZBP1-mediated PANoptosis of NP cells and result in IDD.

### 3.4 Kindlin-2 inhibits mechanical overloading-induced PANoptosis of NP cells

**Figure 5:**
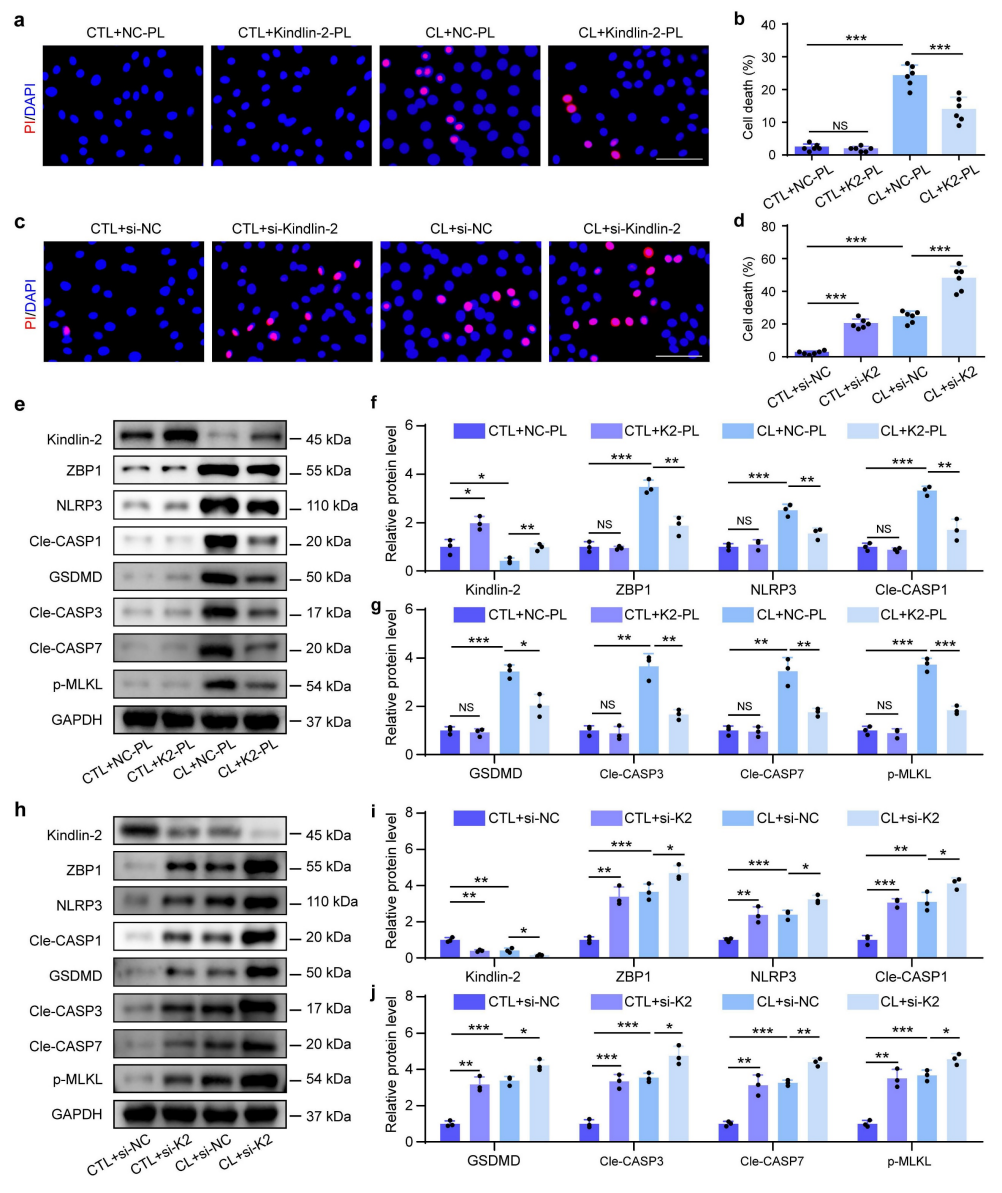
**Kindlin-2 inhibits mechanical overloading-induced PANoptosis of NP cells *in vitro*. (a, b)** Propidium iodide (PI) staining and cell death rate of NP cells, which were transfected with negative control plasmid (NC-PL) or Kindlin-2 plasmid (Kindlin-2-PL), and then treated with or without compression loading (CL) treatment (n=6). Scale bar, 50 μm. **(c, d)** PI staining and cell death rate of NP cells, which were transfected with negative control siRNA (si-NC) or Kindlin-2 siRNA (si-Kindlin-2), and then treated with or without CL treatment (n=6). Scale bar, 50 μm.** (e-g)** Western blotting analyses of Kindlin-2, ZBP1, NLRP3, Cle-CASP1, GSDMD, Cle-CASP3, Cle-CASP7, and p-MLKL of NP cells, which were treated as in (a) (n=3). **(h-j)** Western blotting analyses of Kindlin-2, ZBP1, NLRP3, Cle-CASP1, GSDMD, Cle-CASP3, Cle-CASP7, and p-MLKL of NP cells, which were treated as in (c) (n=3). Results are expressed as mean ± standard deviation (s.d.). NS, no statistical significance, *P < 0.05, **P < 0.01, ***P < 0.001.

Our prior study demonstrated that Kindlin-2, a key focal adhesion protein, protects against mechanical overloading-induced NP cell death and IDD by suppressing NLRP3 inflammasome activation^11^. To investigate its role in mechanical overloading-induced PANoptosis, we modulated Kindlin-2 expression by siRNA-mediated knockdown and plasmid (PL)-based overexpression in NP cells with and without CL. PI staining demonstrated that Kindlin-2 overexpression reduced CL-induced NP cell death (Figure 5a, b). Western blotting revealed that Kindlin-2 overexpression partially reversed the upregulation of the expressions of ZBP1 and PANoptosis-related proteins caused by CL in NP cells, including NLRP3, Cle-CASP1, GSDMD, Cle-CASP3, Cle-CASP7, and p-MLKL (Figure 5e-g). In contrast, Kindlin-2 knockdown promoted NP cell death and could accelerate CL-induced NP cell death (Figure 5c, d). Moreover, Results from western blotting showed that Kindlin-2 knockdown increased the expressions of ZBP1 and PANoptosis-related proteins in NP cells, including NLRP3, Cle-CASP1, GSDMD, Cle-CASP3, Cle-CASP7, and p-MLKL, and could further exacerbate the molecular changes induced by CL (Figure 5h-j). Therefore, our data suggest that Kindlin-2 can inhibit ZBP1-mediated PANoptosis in NP cells under mechanical overloading condition.

### 3.5 Kindlin-2 suppresses PANoptosis by promoting SUMOylation of FOXP1 at K636 in NP cells

**Figure 6:**
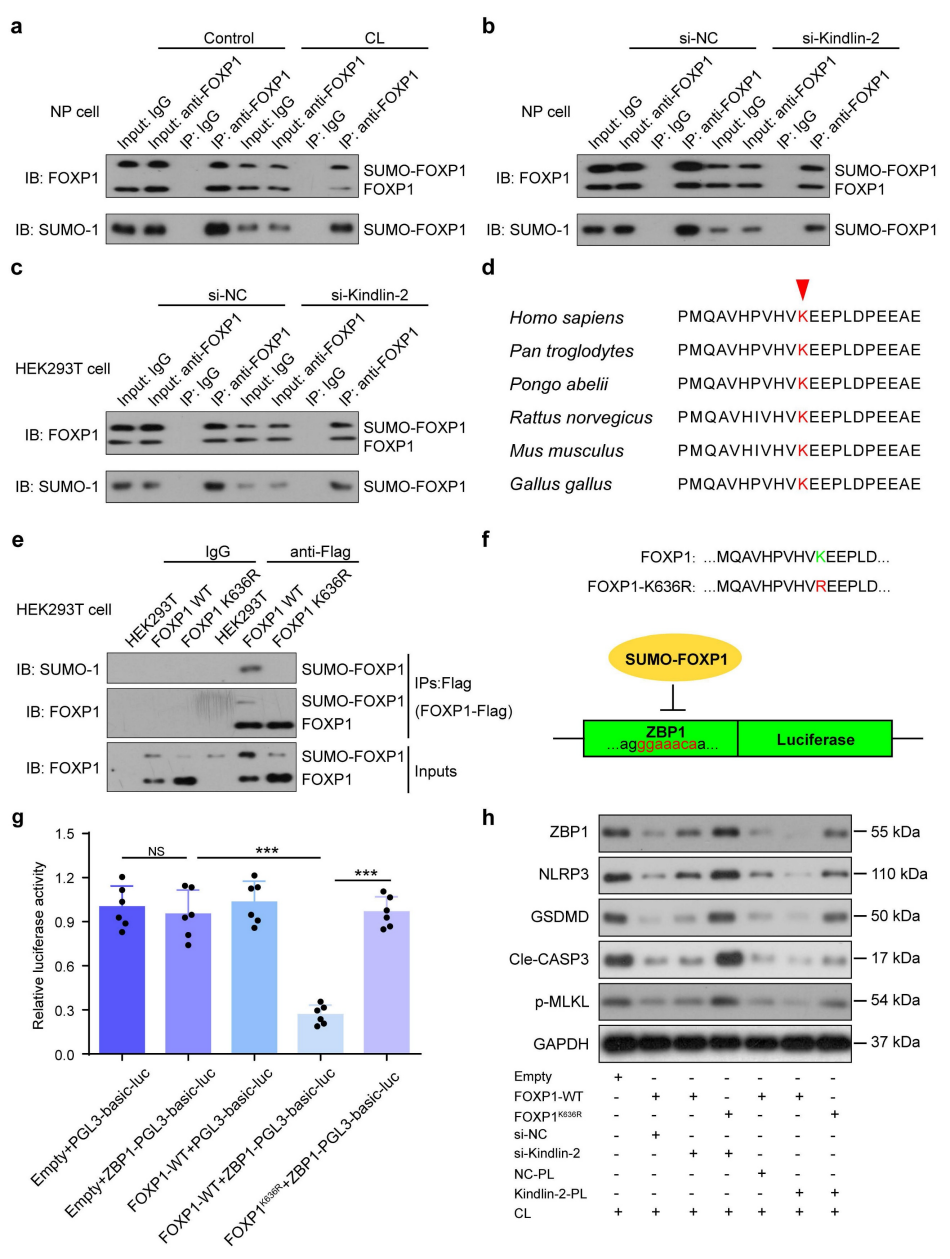
**Kindlin-2 suppresses ZBP1-mediated PANoptosis by promoting SUMOylation of FOXP1 at K636 in NP cells. (a)** Co-immunoprecipitation of FOXP1 and SUMO-1 in NP cells treated with or without compression loading (CL). **(b)** Co-immunoprecipitation of FOXP1 and SUMO-1 in NP cells transfected with negative control siRNA (si-NC) or Kindlin-2 siRNA (si-Kindlin-2). **(c)** Co-immunoprecipitation of FOXP1 and SUMO-1 in HEK293T cells transfected with negative control siRNA (si-NC) or Kindlin-2 siRNA (si-Kindlin-2). **(d)** K636 is conserved across species. **(e)** Co-immunoprecipitation of FOXP1 and SUMO-1 in HEK293T cells transfected with empty, FOXP1 WT, or FOXP1^K636R^ plasmids. **(f, g)** Luciferase activity in HEK293T transfected with empty, pGL3-ZBP1-Luc, FOXP1 WT, or FOXP1^K636R^ plasmids. **(h)** Western blotting analyses of ZBP1, NLRP3, GSDMD, Cle-CASP3, and p-MLKL of NP cells, which were transfected with empty, FOXP1 WT, or FOXP1K636R plasmids, negative control siRNA (si-NC) or Kindlin-2 siRNA (si-Kindlin-2), negative control plasmid (NC-PL) or Kindlin-2 plasmid (Kindlin-2-PL), and then treated with or without compression loading (CL) treatment. NS, no statistical significance, ***P < 0.001.

The Forkhead box (FOX) family of transcription factors regulate diverse biological processes, including cell death, by binding specific DNA sequences and interacting with other co-regulators. This activity is regulated by post-translational modifications, such as SUMOylation. Previous study demonstrated that Kindlin-2 could regulate FOX protein, and FOXP1 was involved in abnormal mechanical stress-induced NLRP3 inflammasome activation^23^, ^26^. Results from the immunoprecipitation (IP) assay demonstrated that mechanical overloading was associated with a reduction in both total FOXP1 protein levels and its SUMOylated form in NP cells (Figure 6a). After transfecting wild-type FOXP1 plasmids into both HEK293T and NP cells, followed by Kindlin-2 knockdown, IP assays revealed that si-Kindlin-2 was also significantly associated with a reduction in both total FOXP1 protein levels and its SUMOylated form in both cell types (Figure 6b, c). K636 was previously identified as an evolutionarily conserved residue and a consensus SUMOylation site in FOXP1 (Figure 6d)^27^. We constructed wild-type and K636R mutant FOXP1 plasmids with Flag tags, which were then respectively transfected into HEK293T cells. IP was performed using IgG and Flag antibodies to assess FOXP1 SUMOylation levels. The results demonstrated that while wild-type FOXP1 showed detectable SUMOylation, the K636R mutant completely lost this modification, suggesting that K636 serves as the major SUMOylation site (Figure 6e). A 200-bp DNA fragment encompassing the predicted FOXP1-binding site in the ZBP1 promoter was cloned into the pGL3-Basic-Luc dual-luciferase reporter vector. HEK293T cells were co-transfected with FOXP1 (wild-type or K636R mutant), ZBP1, and the Renilla luciferase control plasmid (pRL-TK). Dual-luciferase assays revealed that FOXP1-WT significantly repressed ZBP1 promoter activity compared to the control. In contrast, the K636R mutation attenuated this inhibitory effect, resulting in elevated luciferase activity. These results suggest that FOXP1 suppresses ZBP1 transcription and that SUMOylation at K636 is critical for its repressive function (Figure 6f, g). To further investigate the role of FOXP1 in Kindlin-2-mediated suppression of mechanical overloading-induced PANoptosis, Kindlin-2 siRNA or plasmid and wild-type or K636R mutant FOXP1 plasmids were transfected into NP cells with CL. Western blot analysis demonstrated that FOXP1 overexpression suppressed ZBP1-mediated PANoptosis, and this effect was attenuated by Kindlin-2 knockdown. In contrast to FOXP1-WT overexpression, the FOXP1 K636R mutant failed to alleviate ZBP1-mediated PANoptosis induced by Kindlin-2 knockdown. Kindlin-2 overexpression did not further suppress Zbp1-mediated PANoptosis in the presence of FOXP1 overexpression, suggesting that FOXP1 acts downstream of Kindlin-2. Moreover, under conditions of Kindlin-2 overexpression, the FOXP1 K636R mutant failed to further suppress ZBP1-mediated PANoptosis compared with FOXP1-WT, indicating that lysine 636 is required for FOXP1-mediated inhibition of PANoptosis. Results showed that Kindlin-2 suppressed ZBP1-mediated PANoptosis by promoting SUMOylation of FOXP1 at K636 in NP cells exposed to CL (Figure 6h). These results suggest that Kindlin-2 inhibition induced by abnormal mechanical loading can downregulate FOXP1 by impairing SUMOylation of FOXP1 at K636, which promotes ZBP1 transcription and PANoptosis activation in NP cells.

### 3.6 Kindlin-2 deletion accelerates NP cell PANoptosis and IDD in the presence of mechanical overloading in mice

**Figure 7:**
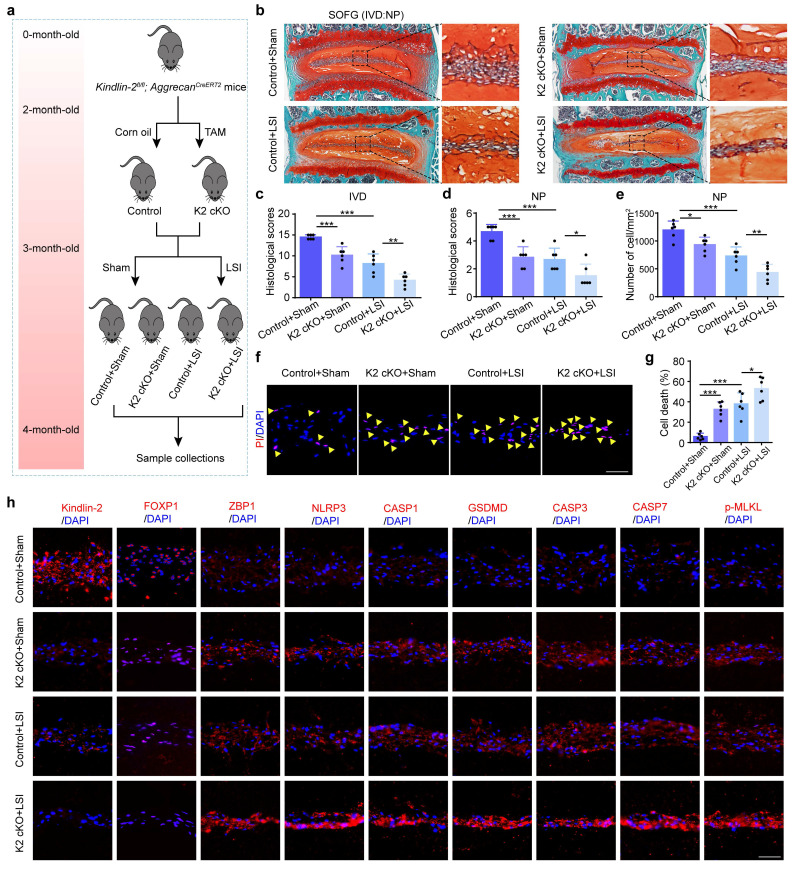
**Kindlin-2 deletion promotes NP cell PANoptosis and IDD in the presence of mechanical overloading in mice. (a)** Breeding strategy and experimental grouping. **(b-d)** SO&FG staining and histological scores of IVD and NP in control and Kindlin-2 cKO mice with or without lumbar spine instability (LSI). (n=6). Scale bar, 100 μm. **(e)** The number of NP cells in mice treated as in (b) (cells/mm^2^, n=6). **(f, g)** Propidium iodide (PI) staining and cell death rate of NP cells in mice treated as in (b) (n=6). Scale bar, 50 μm. **(h)** IF staining of Kindlin-2, FOXP1, ZBP1, NLRP3, CASP1, GSDMD, CASP3, CASP7, and p-MLKL in NP in mice treated as in (b). Scale bar, 50 μm. Results are expressed as mean ± standard deviation (s.d.). *P < 0.05, **P < 0.01, ***P < 0.001.

Above *in vitro* findings suggest that Kindlin-2 plays a critical role in regulating NP cell PANoptosis and IDD, prompting us to validate its function *in vivo*. To this end, we generated conditional knockout mice (K2 cKO) by crossing *Kindlin-2^fl/fl^* mice with *Aggrecan^CreERT2^* mice. Two-month-old male K2 cKO and littermate control mice were treated with tamoxifen (TAM) to delete Kindlin-2 specifically in Aggrecan-expressing cells, while control mice received corn oil. One month post-induction, to mimic mechanical overloading-induced IDD, both K2 cKO and control mice underwent LSI surgery or sham operation. IVD tissues were harvested one month post-surgery for further analysis (Figure 7a). The integration of cKO mice with the LSI model furnishes robust genetic evidence regarding the proposed mechanism under aberrant biomechanical conditions, ensuring rigorous validation from a genetic perspective. Histological assessment using SO&FG staining revealed significantly lower scores in IVDs and NP tissues of K2 cKO mice compared to controls (Figure 7b-d). Consistently, the NP cell number was markedly reduced in K2 cKO mice (Figure 7e). PI staining further confirmed an increased rate of cell death in NP tissues from K2 cKO mice (Figure 7f-g). IF analysis demonstrated downregulation of FOXP1 and upregulation of PANoptosis-associated proteins, including ZBP1, NLRP3, CASP1, GSDMD, CASP3, CASP7, and p-MLKL in K2 cKO mice relative to controls (Figure 7h, Supplementary Figure 2). Furthermore, K2 cKO mice subjected to LSI exhibited exacerbated IDD, as evidenced by significantly lower histological scores compared to LSI control mice. This was accompanied by a more pronounced reduction in NP cell number and a further increase in both cell death rates and expression levels of PANoptosis-related proteins (Figure 7).

### 3.7 Ciliary IFT88 regulates the expression of Kindlin-2 in NP cells under mechanical overloading condition

**Figure 8:**
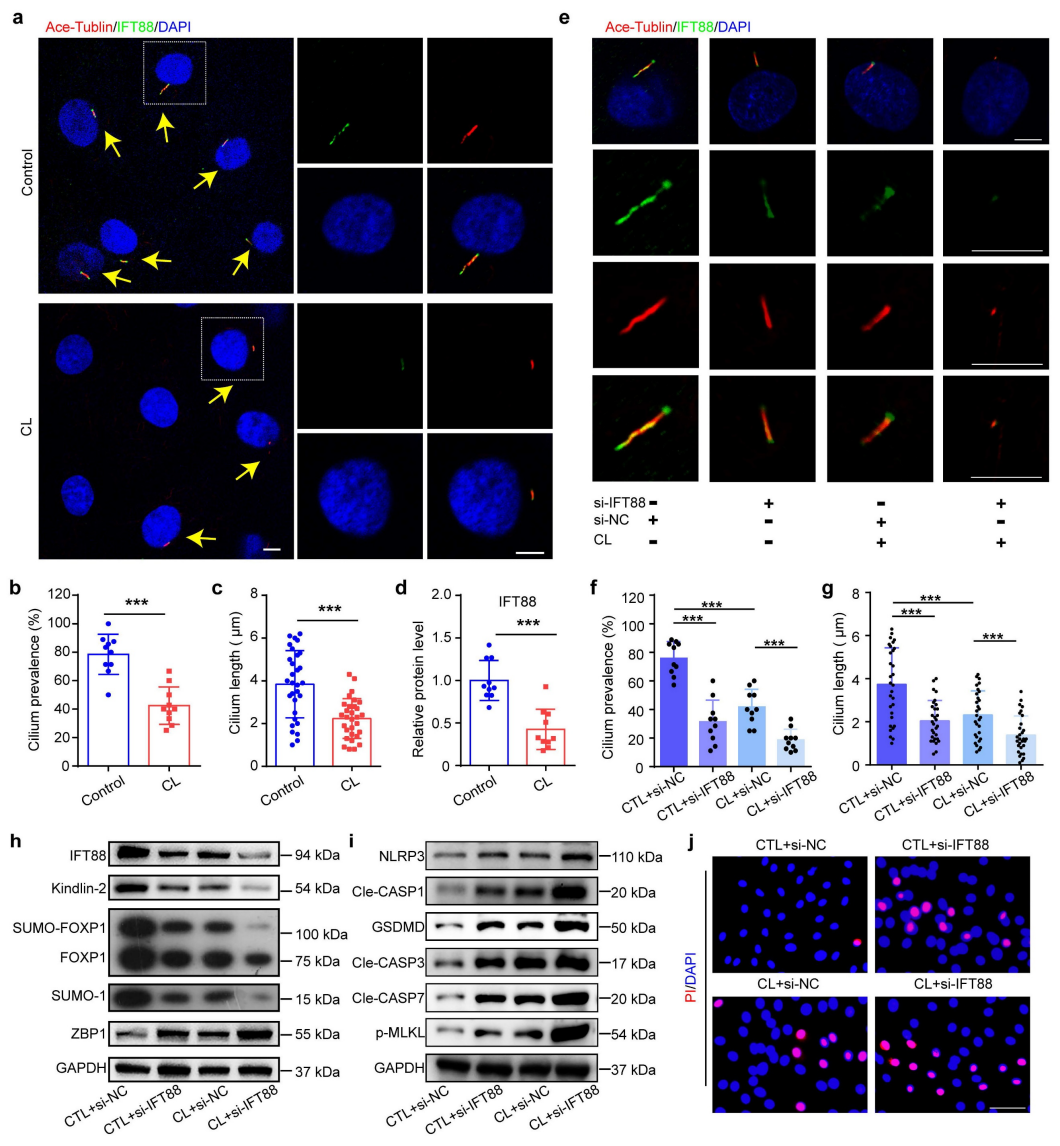
**IFT88 inhibition decreases Kindlin-2 expression and promotes PANoptosis of NP cells under mechanical overloading *in vitro*. (a)** IF staining of Ace-Tublin and IFT88 in NP cells treated with or without compression loading (CL). Yellow arrows represent primary cilia. Scale bar, 5 μm. **(b, c)** Cilium prevalence (n=10) and length (n=30) in NP cells treated as in (a). **(d)** Relative protein level of IFT88 in NP cells treated as in (a) (n=10). **(e)** IF staining of Ace-Tublin and IFT88 in NP cells, which were transfected with negative control siRNA (si-NC) or IFT88 siRNA (si-IFT88), and then treated with or without CL treatment (n=6). Scale bar, 5 μm. **(f, g)** Cilium prevalence (n=10) and length (n=30) in NP cells treated as in (e). **(h, i)** Western blotting analyses of IFT88, Kindlin-2, FOXP1, SUMO-1, ZBP1, NLRP3, Cle-CASP1, GSDMD, Cle-CASP3, Cle-CASP7, and p-MLKL of NP cells, which were treated as in (e) (n=3). **(j)** Propidium iodide (PI) staining of NP cells treated as in (e). Scale bar, 50 μm. Results are expressed as mean ± standard deviation (s.d.). ***P < 0.001.

**Figure 9:**
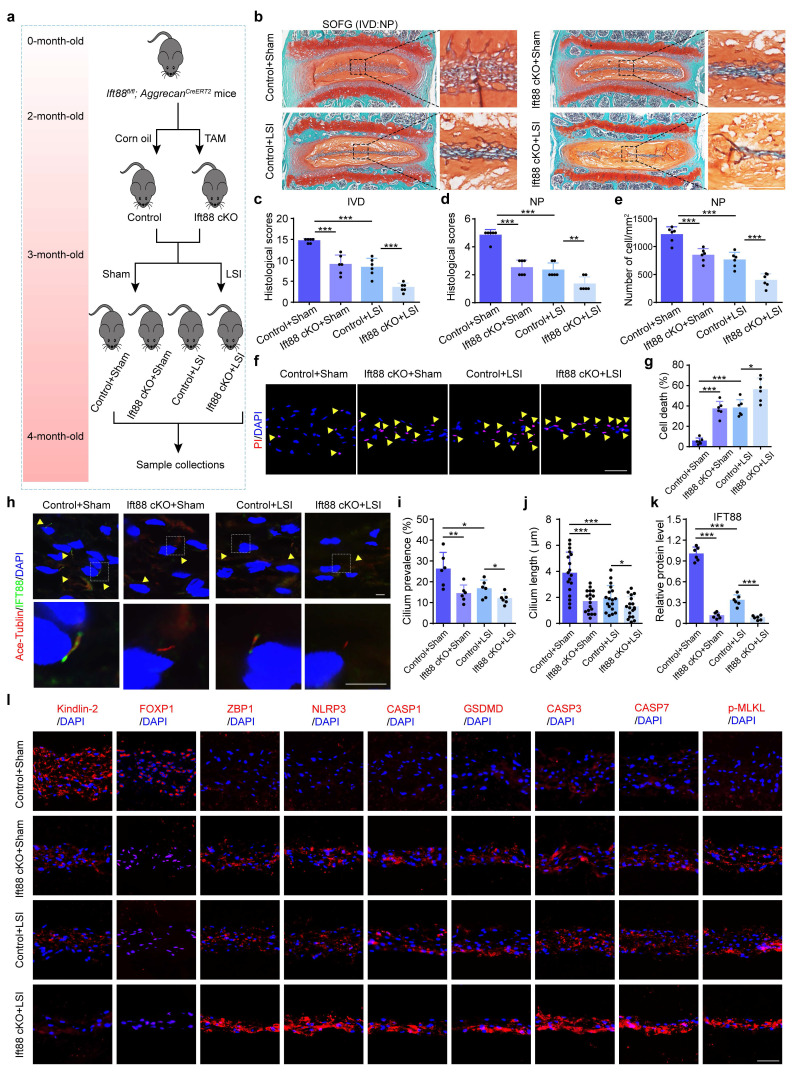
** IFT88 deletion decreases Kindlin-2 expression, promotes NP cell PANoptosis and IDD in the presence of mechanical overloading in mice. (a)** Breeding strategy and experimental grouping. **(b-d)** SO&FG staining and histological scores of IVD and NP in control and Ift88 cKO mice with or without lumbar spine instability (LSI). (n=6). Scale bar, 100 μm. **(e)** The number of NP cells in mice treated as in (b) (cells/mm^2^, n=6). **(f, g)** Propidium iodide (PI) staining and cell death rate of NP cells in mice treated as in (b) (n=6). Scale bar, 50 μm. **(h)** IF staining of Ace-Tublin and IFT88 in NP cells in mice treated as in (b). Yellow arrows represent primary cilia. Scale bar, 5 μm. **(i, j)** Cilium prevalence (n=6) and length (n=18) in NP cells treated as in (a).** (k)** Relative protein level of IFT88 (n=6). **(l)** IF staining of Kindlin-2, FOXP1, ZBP1, NLRP3, CASP1, GSDMD, CASP3, CASP7, and p-MLKL in NP in mice treated as in (b). Scale bar, 50 μm. Results are expressed as mean ± standard deviation (s.d.). *P < 0.05, **P < 0.01, ***P < 0.001.

The molecular mechanisms by which NP cells sense mechanical overloading and transduce these signals through Kindlin-2-mediated downstream signals remain unclear. Previous studies have suggested that ciliary IFT88 may play a pivotal role in mechanosensation within NP cells^28^. IF staining showed that CL inhibited cilium prevalence, cilium length, and IFT88 expression level (Figure 8a-d), and IFT88 knockdown using IFT88 siRNA could further exacerbate these changes (Figure 8e-g). Results from western blotting demonstrated that si-IFT88 decreased Kindlin-2 expression and FOXP1 SUMOylation, upregulated the expressions of ZBP1 and PANoptosis-related protein, including NLRP3, Cle-CASP1, GSDMD, Cle-CASP3, Cle-CASP7, and p-MLKL (Figure 8h, i). And PI staining revealed that si-IFT88 promoted NP cell death (Figure 8j). Moreover, si-IFT88 could further exacerbate CL-induced decreased expression of Kindlin-2 and PANoptosis of NP cells (Figure 8h-j). In addition, we generated conditional knockout mice (Ift88 cKO) by crossing *Ift88^fl/fl^* mice with *Aggrecan^CreERT2^* mice, and applied LSI model to mimic mechanical overloading (Figure 9a). SO&FG staining demonstrated that the histological scores in IVDs and NP tissues were significantly lower in Ift88 cKO mice than in control mice (Figure 9b-d). Correspondingly, a notable decrease in the NP cell number was observed in Ift88 cKO mice (Figure 9e). PI staining provided additional confirmation of an elevated cell death rate in NP tissues from Ift88 cKO mice (Figure 9f-g). IF analysis revealed reduced cilium prevalence, cilium length, and decreased Kindlin-2 and FOXP1 expression, and upregulated expression of PANoptosis-associated proteins, including ZBP1, NLRP3, CASP1, GSDMD, CASP3, CASP7, and p-MLKL, in Ift88 cKO mice compared to controls (Figure 9h-l, Supplementary Figure 3). Moreover, when subjected to LSI, Ift88 cKO mice exhibited aggravated IDD, as indicated by significantly lower histological scores than those in LSI control mice. This deterioration was paralleled by a more substantial decline in NP cell numbers and further increases in both cell death rates and the expression levels of PANoptosis-related proteins (Figure 9, Supplementary Figure 3). Thus, our results indicate that ciliary IFT88 deficiency downregulates Kindlin-2 expression and induces ZBP1-mediated PANoptosis in NP cells under mechanical overloading condition.

## 4. Discussion

In this study, we elucidate a novel molecular mechanism by which mechanical overloading induces IDD through ZBP1-mediated PANoptosis in NP cells. PANoptosis has also been implicated in other musculoskeletal degenerative diseases, such as osteoarthritis and osteoporosis^29^, ^30^. However, the regulatory networks governing PANoptosis exhibit marked tissue specificity. Specifically, NP cells reside in a unique microenvironment characterized by persistent and complex biomechanical stress. Rather than relying solely on biochemical inflammatory signals, NP cells utilize the primary ciliary protein IFT88 as a major mechanosensor to perceive mechanical overloading. The IFT88/Kindlin-2/FOXP1 mechanosensitive axis thus represents a novel, NP-specific PANoptosis regulatory paradigm that fundamentally distinguishes the pathogenesis of IDD from other musculoskeletal degenerative. Our comprehensive investigations reveal that abnormal mechanical stress disrupts ciliary IFT88 signaling, leading to marked downregulation of the key focal adhesion protein Kindlin-2. The reduction in Kindlin-2 expression subsequently impairs SUMOylation of the transcription factor FOXP1 at K636, which in turn triggers significant upregulation of ZBP1 expression. This molecular cascade ultimately activates the PANoptosis pathway, a newly characterized programmed cell death modality integrating features of pyroptosis, apoptosis, and necroptosis, in NP cells, thereby accelerating IDD progression. Beyond providing crucial insights into the mechanobiology of IDD, the discovery of this pathway establishes a conceptual advance with profound translational relevance. It shifts the therapeutic paradigm from targeting single, isolated death mechanisms to a multi-pronged approach, wherein modulating upstream mechanosensors IFT88/Kindlin-2 or master sensors ZBP1 can comprehensively rescue NP cells from the interconnected web of PANoptosis.

The lumbar spine, which supports upright posture and facilitates upper body movement, is subjected to substantial mechanical forces generated by back musculature, emphasizing the critical role of mechanical stress in IVD homeostasis and disease. IVD is a mechanically loaded tissue, wherein cells are continuously exposed to complex biomechanical stresses, including compression, tension, and shear forces^31^. These mechanical stimuli play a pivotal role in regulating cellular metabolism and viability within the disc matrix. In particular, the gelatinous NP, located centrally within the IVD, is essential for maintaining proper disc biomechanics. Notably, excessive mechanical loading can induce NP cell death, a key pathological feature associated with IDD. Substantial experimental evidence has demonstrated that multiple regulated cell death modes, including pyroptosis^8^, apoptosis^32^, and necroptosis^33^, contribute to mechanical overloading-induced NP cell death. Recently, increasing evidences have demonstrated extensive crosstalk among pyroptosis, apoptosis, and necroptosis^34^-^36^, suggesting the existence of potential core regulatory mechanisms that may coordinately govern these three cell death modalities in NP cell death. PANoptosis is a newly identified form of regulated cell death, which encompasses pivotal molecular components from pyroptosis, apoptosis, and necroptosis pathways. Therefore, we speculate that PANoptosis is involved in mechanical overloading-induced NP cell death and plays a critical role in IDD. To verify our hypothesis, we first used human degenerated IVD tissues and aged mice to assess PANoptosis in NP cells. We observed that the expression of PANoptosis-related proteins and cell death in NP tissues progressively increased with the severity of IDD in both human and mice. The IVD microenvironment is highly complex, encompassing mechanical overloading, acidity, hypertonicity, nutrient deficiency, and hypoxia^37^. To specifically investigate whether mechanical overloading induces PANoptosis in NP cells, we applied LSI mouse model and an *in vitro* mechanical compression system to simulate pathological loading conditions. We found that mechanical overloading upregulated the expression levels of PANoptosis-related proteins and promoted cell death. Our results demonstrate that PANoptosis is involved in IDD and mechanical overloading can induce NP cell PANoptosis.

The initiation of PANoptosis is dependent on the activation and upregulation of sensor proteins in response to adverse microenvironmental conditions^15^. ZBP1 is a well-established regulator of NLRP3 inflammasome activation, and is also characterized as a key innate sensor protein and plays an essential role in the initiation of the PANoptosis^38^. Our results showed that the expressions of ZBP1 in NP tissues progressively increased with the severity of IDD in both human and mice, and it could be upregulated by mechanical overloading in NP cells. Moreover, we found that ZBP1 inhibition alleviated NP cell PANoptosis and IDD under mechanical overloading conditions both *in vitro* and *in vivo*. These findings suggest that PANoptosis significantly contributes to the pathogenesis of IDD, and mechanical overloading can activate ZBP1-mediated PANoptosis of NP cells and result in IDD. Notably, in addition to ZBP1, distinct environmental stimuli in different cells can activate and upregulate other specific PANoptosis sensors, such as NLRP12 and absent in melanoma 2 (AIM2), thereby initiating PANoptosis^39^. However, it remains unclear whether these sensors can also respond to mechanical loading to induce PANoptosis in NP cells and IDD, and the underlying molecular mechanisms require further investigation.

Kindlin-2, a crucial adhesion-associated molecule of the Kindlin family^40^-^42^, plays pivotal roles in regulating diverse pathophysiological processes and has been implicated in various diseases including cancer, cardiovascular disorders, gastrointestinal diseases, and particularly musculoskeletal disorders^43^-^46^. Our group has generated multiple Kindlin-2 cKO mouse models, which have demonstrated its essential function in mechanotransduction and the maintenance of bone and cartilage homeostasis^47^-^49^. Most recently, we discovered that Kindlin-2 protects against IDD by suppressing NLRP3 inflammasome activation and subsequent apoptosis of NP cells under pathological mechanical stress^11^. These findings suggest that Kindlin-2 may play a critical role in mechanical overloading-induced PANoptosis of NP cells. In this study, we employed both gain- and loss-of-function approaches, utilizing siRNA-mediated knockdown and plasmid overexpression *in vitro*, along with conditional knockout mice *in vivo*, to investigate Kindlin-2 function. Our findings demonstrate that Kindlin-2 protects against mechanical overload-induced IDD by suppressing ZBP1-mediated PANoptosis in NP cells. Further mechanistic analysis reveals that Kindlin-2 induces FOXP1 SUMOylation at K636, thereby inhibiting ZBP1 expression and subsequent PANoptosis in NP cells. In addition, results shown that Kindlin-2 overexpression further decreases ZBP1 despite the FOXP1 K636 mutation. This observation may be explained by residual endogenous WT FOXP1 expression in NP cells. However, the precise mechanosensing mechanisms by which NP cells detect mechanical overloading and transduce these signals through Kindlin-2-mediated signaling cascades have yet to be fully elucidated.

Primary cilium is a highly conserved microtubule-based organelle projecting from the cell membrane into the extracellular environment^50^, ^51^. Long considered a vestigial organ, emerging evidence now establishes its critical role in mechanotransduction and pathophysiology of the musculoskeletal system^52^, ^53^. Primary cilia assembly depends on intraflagellar transport proteins such as IFT88^54^, ^55^. Recent studies demonstrate that conditional knockout of IFT88 in postnatal murine chondrocytes leads to progressive cartilage thinning and accelerates stress-induced osteoarthritis progression^56^, ^57^. Our published work reveals that IFT88 deletion impairs primary cilia formation and attenuates fluid shear stress-induced extracellular matrix synthesis in NP cells^28^. Additional studies show that IFT88 ablation in murine intervertebral discs promotes disc degeneration by suppressing parathyroid hormone type 1 receptor (PTH1R) signaling^58^. Notably, our previous research identified Kindlin-2 as a functional component of PTH1R signaling in bone homeostasis^47^. Based on these findings, we hypothesize that primary cilia serve as mechanosensors for pathological mechanical stress in NP cells and mediate Kindlin-2-dependent downstream signaling. To verify our hypothesis, we utilized IFT88 siRNA-mediated knockdown and generated Ift88 cKO mice. Our findings demonstrate that mechanical overloading suppresses ciliary IFT88 signaling, resulting in decreased Kindlin-2 expression and subsequent ZBP1-mediated PANoptosis in NP cells, ultimately contributing to IDD.

There are several limitations to this study. First, the mechanism underlying the downregulation of FOXP1 expression was not clearly elucidated, which may be attributable to reduced SUMOylation of FOXP1. Future studies utilizing techniques such as *in vivo* SUMOylation assays in the presence of proteasome inhibitors are needed to clarify the direct regulation of FOXP1 SUMOylation under mechanical stress. Further investigation is required to determine the extent to which Kindlin-2 deletion affects FOXP1 expression and its post-translational modifications. Second, gain-of-function experiments were not conducted to definitively establish a direct regulatory relationship between ciliary IFT88 and Kindlin-2. Third, although we mechanistically linked the IFT88/Kindlin-2/FOXP1 axis to ZBP1-mediated PANoptosis suppression, therapeutic targeting of this pathway remains unexplored. Future studies will focus on the screening and development of small-molecule compounds that enhance FOXP1 SUMOylation or directly inhibit ZBP1 activation. Evaluating the therapeutic efficacy of such targeted drug interventions combined with biomaterial-based local delivery systems in IDD animal models represents a critical step toward translating our findings into clinical applications. As the largest avascular organ in the human body, the intervertebral disc is characterized by structural properties that severely restrict drug penetration and local retention following systemic administration. Although PANoptosis inhibition exerts cytoprotective effects on NP cells under mechanical overload conditions, systemic blockade of this innate immune and cell death pathway may inadvertently compromise normal host defense mechanisms and cellular turnover in healthy tissues. Therefore, the development of strictly localized delivery strategies or disc-specific targeting molecules is imperative to maximize therapeutic efficacy while minimizing systemic off-target toxicity. Fourth, while the inhibition of ZBP1 significantly attenuated PANoptosis markers, the residual cell death observed suggests that ZBP1 is not the sole executor in this process. This raises the intriguing possibility that upstream regulators, such as the IFT88/Kindlin-2 signaling axis, may orchestrate a broader cell death landscape. Beyond its potential role in ZBP1-mediated PANoptosis, the IFT88/Kindlin-2 signaling axis may represent a broader regulatory hub that crosstalks with other regulated cell death forms (e.g., ferroptosis) and canonical IDD-related pathways (e.g., TGF-β, Wnt/β-catenin, NF-κB) to orchestrate the complex pathogenesis of IDD. This study also did not explore the potential role of the IFT88/Kindlin-2/FOXP1 axis in regulating extracellular matrix anabolism and catabolism. Unraveling these complex networks will be crucial for developing effective therapeutic strategies. Systematic investigation of these interactions could reveal broader therapeutic opportunities. Fifth, although PI staining has been widely employed to quantify nucleus pulposus cell death both *in vitro* and *in vivo*, it serves as a general marker of late-stage membrane permeability changes and cannot distinctly differentiate PANoptosis from specific cell death pathways. Accordingly, our conclusions regarding PANoptosis in NP cells are primarily based on the concomitant upregulation of specific molecular effectors observed at both molecular and morphological levels. At last, as most data were derived from rodent models, translational relevance requires validation in higher-order species. We are designing follow-up studies to examine IFT88/Kindlin-2-regulated PANoptosis in non-human primates, which better recapitulate human disc pathophysiology. And we state that the Aggrecan-CreERT2 driver is active in multiple cartilage-lineage tissue. Given that our phenotypic analyses were predominantly focused on and rigorously quantified within the NP tissue, the observed degenerative phenotypes are most likely attributable to the NP-specific deletion of our target gene.

## 5. Conclusion

In conclusion, we demonstrate that IFT88/Kindlin-2 signaling protects against IDD by preventing mechanical overloading-induced PANoptosis of NP cells via FOXP1 SUMOylation activation. This work provides novel insights into the molecular pathogenesis of IDD and suggests potential therapeutic targets for its treatment.

## Supplementary Material

Supplementary figures and tables.

## Data Availability

The data that support the findings of this study are available from the corresponding authors upon reasonable request.

## Abbreviations

IVD: intervertebral disc
IDD: intervertebral disc degeneration
NP: nucleus pulposus
ZBP1: Z-DNA-binding protein 1
FOXP1: forkhead box P1
IFT88: intraflagellar transport 88
LBP: low back pain
RIPK: receptor-interacting protein kinase
FADD: Fas-associated protein with death domain
CASP: caspase
NLRP3: NOD-like receptor family pyrin domain-containing 3
ASC: apoptosis-associated speck-like protein containing a CARD
MLKL: mixed lineage kinase domain-like protein
GSDME: gasdermin E
LSI: lumbar spine instability
CIC: coccygeal IVD compression
NC: negative control
H&E: hematoxylin and eosin
SO/FG: safranin O/fast green
IF: immunofluorescence
PI: Propidium iodide
CL: compression loading
cKO: conditional knockout
TAM: tamoxifen
IP: immunoprecipitation
PL: plasmid
WT: wild-type

## References

[1] Lytras D (2025). Towards sustainable relief in chronic low back pain. The Lancet Rheumatology.

[2] Chen S, Chen M, Wu X, Lin S, Tao C, Cao H (2022). Global, regional and national burden of low back pain 1990-2019: A systematic analysis of the Global Burden of Disease study 2019. J Orthop Translat.

[3] Lin Z, Wang H, Song J, Xu G, Lu F, Ma X (2023). The role of mitochondrial fission in intervertebral disc degeneration. Osteoarthritis Cartilage.

[4] Ogaili RH, Alassal A, Za'aba NF, Zulkiflee I, Mohd Isa IL (2025). Regenerative strategies for intervertebral disc degeneration. J Orthop Translat.

[5] Liu F, Chao S, Yang L, Chen C, Huang W, Chen F (2024). Molecular mechanism of mechanical pressure induced changes in the microenvironment of intervertebral disc degeneration. Inflammation research.

[6] Chen S, Zhao L, Deng X, Shi D, Wu F, Liang H (2017). Mesenchymal stem cells protect nucleus pulposus cells from compression-induced apoptosis by inhibiting the mitochondrial pathway. Stem Cells Int.

[7] Chen S, Lv X, Hu B, Shao Z, Wang B, Ma K (2017). RIPK1/RIPK3/MLKL-mediated necroptosis contributes to compression-induced rat nucleus pulposus cells death. Apoptosis.

[8] Fu F, Bao R, Yao S, Zhou C, Luo H, Zhang Z (2021). Aberrant spinal mechanical loading stress triggers intervertebral disc degeneration by inducing pyroptosis and nerve ingrowth. Sci Rep.

[9] Wu Q, He X, Wu LM, Zhang RY, Li LM, Wu CM (2020). MLKL aggravates Ox-LDL-induced cell pyroptosis via activation of NLRP3 inflammasome in human umbilical vein endothelial cells. Inflammation.

[10] Lin Q, Li S, Jiang N, Jin H, Shao X, Zhu X (2021). Inhibiting NLRP3 inflammasome attenuates apoptosis in contrast-induced acute kidney injury through the upregulation of HIF1A and BNIP3-mediated mitophagy. Autophagy.

[11] Chen S, Wu X, Lai Y, Chen D, Bai X, Liu S (2022). Kindlin-2 inhibits Nlrp3 inflammasome activation in nucleus pulposus to maintain homeostasis of the intervertebral disc. Bone Res.

[12] Malireddi RKS, Kesavardhana S, Kanneganti TD (2019). ZBP1 and TAK1: Master regulators of NLRP3 inflammasome/pyroptosis, apoptosis, and necroptosis (PAN-optosis). Front Cell Infect Microbiol.

[13] Lin JF, Wang TT, Huang RZ, Tan YT, Chen DL, Ju HQ (2025). PANoptosis in cancer: bridging molecular mechanisms to therapeutic innovations. Cell Mol Immunol.

[14] Wang S, Song A, Xie J, Wang YY, Wang WD, Zhang MJ (2024). Fn-OMV potentiates ZBP1-mediated PANoptosis triggered by oncolytic HSV-1 to fuel antitumor immunity. Nat Commun.

[15] Pandeya A, Kanneganti TD (2024). Therapeutic potential of PANoptosis: innate sensors, inflammasomes, and RIPKs in PANoptosomes. Trends Mol Med.

[16] Gao X, Ma C, Liang S, Chen M, He Y, Lei W (2024). PANoptosis: Novel insight into regulated cell death and its potential role in cardiovascular diseases (Review). Int J Mol Med.

[17] Li P, Gao Y, Tao Z, Mu Z, Du S, Zhao X (2025). PANoptosis: Cross-Talk Among Apoptosis, Necroptosis, and Pyroptosis in Neurological Disorders. Journal of inflammation research.

[18] Zhu Z, Kong F, Jiang F, Jiang J, Quan D, Guo J (2025). RIPK1-targeted therapy alleviates intervertebral disc degeneration via inhibiting nucleus pulposus PANoptosis. Apoptosis.

[19] Wang K, Ji H, Gao J, Wang Z, Gao Y, Lian X (2026). DDIT3 drives nucleus pulposus cell PANoptosis and intervertebral disc degeneration progression. Apoptosis.

[20] Wu C, Jiao H, Lai Y, Zheng W, Chen K, Qu H (2015). Kindlin-2 controls TGF-beta signalling and Sox9 expression to regulate chondrogenesis. Nat Commun.

[21] Bian Q, Ma L, Jain A, Crane JL, Kebaish K, Wan M (2017). Mechanosignaling activation of TGFβ maintains intervertebral disc homeostasis. Bone Res.

[22] Liao Z, Luo R, Li G, Song Y, Zhan S, Zhao K (2019). Exosomes from mesenchymal stem cells modulate endoplasmic reticulum stress to protect against nucleus pulposus cell death and ameliorate intervertebral disc degeneration in vivo. Theranostics.

[23] Gao H, Zhou L, Zhong Y, Ding Z, Lin S, Hou X (2022). Kindlin-2 haploinsufficiency protects against fatty liver by targeting Foxo1 in mice. Nat Commun.

[24] Yan WT, Zhao WJ, Hu XM, Ban XX, Ning WY, Wan H (2023). PANoptosis-like cell death in ischemia/reperfusion injury of retinal neurons. Neural regeneration research.

[25] Ye D, Xu Y, Shi Y, Fan M, Lu P, Bai X (2022). Anti-PANoptosis is involved in neuroprotective effects of melatonin in acute ocular hypertension model. Journal of Pineal Research.

[26] Zhuang T, Liu J, Chen X, Zhang L, Pi J, Sun H (2019). Endothelial Foxp1 Suppresses Atherosclerosis via Modulation of Nlrp3 Inflammasome Activation. Circ Res.

[27] Usui N, Araujo DJ, Kulkarni A, Co M, Ellegood J, Harper M (2017). Foxp1 regulation of neonatal vocalizations via cortical development. Genes Dev.

[28] Chen S, Qin L, Wu X, Fu X, Lin S, Chen D (2020). Moderate fluid shear stress regulates heme oxygenase-1 expression to promote autophagy and ECM homeostasis in the nucleus pulposus cells. Front Cell Dev Biol.

[29] Zhou D, Luo Y, Li F, Liu T, Mei Y, Li F (2025). Exploring the mechanisms of PANoptosis in osteoarthritis and the therapeutic potential of andrographolide through bioinformatics and single-cell analysis. Biology direct.

[30] Hu HT, Zhang ZY, Luo Z, Ti HB, Wu JJ, Nie H (2025). Emerging regulated cell death mechanisms in bone remodeling: decoding ferroptosis, cuproptosis, disulfidptosis, and PANoptosis as therapeutic targets for skeletal disorders. Cell death discovery.

[31] Neidlinger-Wilke C, Galbusera F, Pratsinis H, Mavrogonatou E, Mietsch A, Kletsas D (2014). Mechanical loading of the intervertebral disc: from the macroscopic to the cellular level. Eur Spine J.

[32] Mao J, Wang D, Wang D, Wu Q, Shang Q, Gao C (2023). SIRT5-related desuccinylation modification of AIFM1 protects against compression-induced intervertebral disc degeneration by regulating mitochondrial homeostasis. Exp Mol Med.

[33] Chen S, Lv X, Hu B, Zhao L, Li S, Li Z (2018). Critical contribution of RIPK1 mediated mitochondrial dysfunction and oxidative stress to compression-induced rat nucleus pulposus cells necroptosis and apoptosis. Apoptosis.

[34] Rojas-Rivera D, Beltrán S, Muñoz-Carvajal F, Ahumada-Montalva P, Abarzúa L, Gomez L (2024). The autophagy protein RUBCNL/PACER represses RIPK1 kinase-dependent apoptosis and necroptosis. Autophagy.

[35] Zheng Z, Deng W, Bai Y, Miao R, Mei S, Zhang Z (2021). The Lysosomal Rag-Ragulator Complex Licenses RIPK1 and Caspase-8-mediated Pyroptosis by Yersinia. Science.

[36] You Z, Huang X, Xiang Y, Dai J, Xu L, Jiang J (2023). Ablation of NLRP3 inflammasome attenuates muscle atrophy via inhibiting pyroptosis, proteolysis and apoptosis following denervation. Theranostics.

[37] Dou Y, Zhang Y, Liu Y, Sun X, Liu X, Li B (2025). Role of macrophage in intervertebral disc degeneration. Bone Res.

[38] Lou J, Mao Y, Jiang W, Shen H, Fan Y, Yu Q (2024). TRIM56 Modulates YBX1 Degradation to Ameliorate ZBP1-Mediated Neuronal PANoptosis in Spinal Cord Injury. Adv Sci (Weinh).

[39] Jin X, Zhu Y, Xing L, Ding X, Liu Z (2025). PANoptosis: a potential target of atherosclerotic cardiovascular disease. Apoptosis.

[40] Wang Y, Huang J, Lin S, Qin L, Hao D, Zhang P (2025). Osteocytic vinculin controls bone mass by modulating Mef2c-driven sclerostin expression in mice. Bone Res.

[41] Huang J, Qin L, Wang Y, Yan Q, Yang H, Chen H (2026). The talin1-p53 axis inhibits osteocyte senescence to promote bone mass and mediate skeletal adaptation to mechanical stimulation. Theranostics.

[42] Lin S, Tao C, Wang Y, Li J, Shi Y, Yan Q (2026). The vinculin-beta-catenin axis promotes bone formation and repair: an essential prerequisite for the anti-osteoporotic efficacy of sclerostin-neutralizing antibody. Science China Life sciences.

[43] Ma N, Wu F, Liu J, Wu Z, Wang L, Li B (2024). Kindlin-2 Phase Separation in Response to Flow Controls Vascular Stability. Circ Res.

[44] Tang W, Ding Z, Gao H, Yan Q, Liu J, Han Y (2023). Targeting Kindlin-2 in adipocytes increases bone mass through inhibiting FAS/PPARγ/FABP4 signaling in mice. Acta Pharmaceutica Sinica B.

[45] Bialkowska K, El Khalki L, Rana PS, Wang W, Lindner DJ, Parker Y (2024). Role of Kindlin 2 in prostate cancer. Sci Rep.

[46] Wu X, Qu M, Gong W, Zhou C, Lai Y, Xiao G (2022). Kindlin-2 deletion in osteoprogenitors causes severe chondrodysplasia and low-turnover osteopenia in mice. J Orthop Translat.

[47] Fu X, Zhou B, Yan Q, Tao C, Qin L, Wu X (2020). Kindlin-2 regulates skeletal homeostasis by modulating PTH1R in mice. Signal Transduct Target Ther.

[48] Cao H, Yan Q, Wang D, Lai Y, Zhou B, Zhang Q (2020). Focal adhesion protein Kindlin-2 regulates bone homeostasis in mice. Bone Research.

[49] Wu X, Lai Y, Chen S, Zhou C, Tao C, Fu X (2022). Kindlin-2 preserves integrity of the articular cartilage to protect against osteoarthritis. Nat Aging.

[50] Lacey SE, Pigino G (2025). The intraflagellar transport cycle. Nat Rev Mol Cell Biol.

[51] Volos P, Fujise K, Rafiq NM (2025). Roles for primary cilia in synapses and neurological disorders. Trends in cell biology.

[52] Lian F, Li H, Ma Y, Zhou R, Wu W (2023). Recent advances in primary cilia in bone metabolism. Frontiers in endocrinology.

[53] Wang T, Chen Y, Zhu X, Zheng L, Li Y, Ruan X (2025). IFT80 and TRPA1 cooperatively regulate bone formation by calcium signaling in response to mechanical stimuli. Metabolism.

[54] Schneider P, Fandrey J, Leu T (2025). Primary cilia as antennas for oxygen. American journal of physiology Cell physiology.

[55] Hilgendorf KI, Myers BR, Reiter JF (2024). Emerging mechanistic understanding of cilia function in cellular signalling. Nat Rev Mol Cell Biol.

[56] Coveney CR, Zhu L, Miotla-Zarebska J, Stott B, Parisi I, Batchelor V (2022). Role of ciliary protein intraflagellar transport protein 88 in the regulation of cartilage thickness and osteoarthritis development in mice. Arthritis Rheumatol.

[57] Chen S, Chen C, Chen M, Li F, Xie C, Shao Z (2025). Roles of primary cilia in cell death. Oral Science and Homeostatic Medicine.

[58] Zheng L, Cao Y, Ni S, Qi H, Ling Z, Xu X (2018). Ciliary parathyroid hormone signaling activates transforming growth factor-beta to maintain intervertebral disc homeostasis during aging. Bone Res.

