# Supplementary material for: IFT88/Kindlin-2 Signaling Prevents Mechanical Overloading-Induced PANoptosis of Nucleus Pulposus Cells by Activating FOXP1 SUMOylation
Source: Int J Biol Sci. 2026 Apr 16;22(9):4598–617. doi: 10.7150/ijbs.132842 (PMC13182237; doi:10.7150/ijbs.132842)
Supplement: Supplementary file 1 — Supplementary figures and tables. [file ijbsv22p4598s1.pdf]

Supplementary Figures

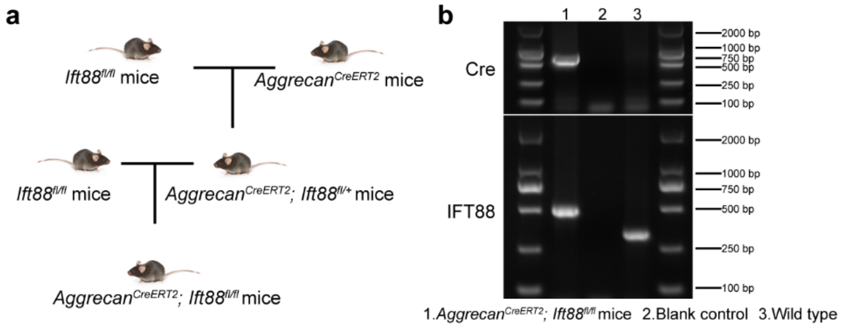

Supplementary Figure 1. Breeding strategy and genotyping. (a) Breeding strategy. (b) Genotyping using tail DNA. IFT88 floxed band: ~500bp; AggrecanCreERT2 band: ~650bp. Primer sequences used in PCR genotyping are listed in Supplementary Table 2.

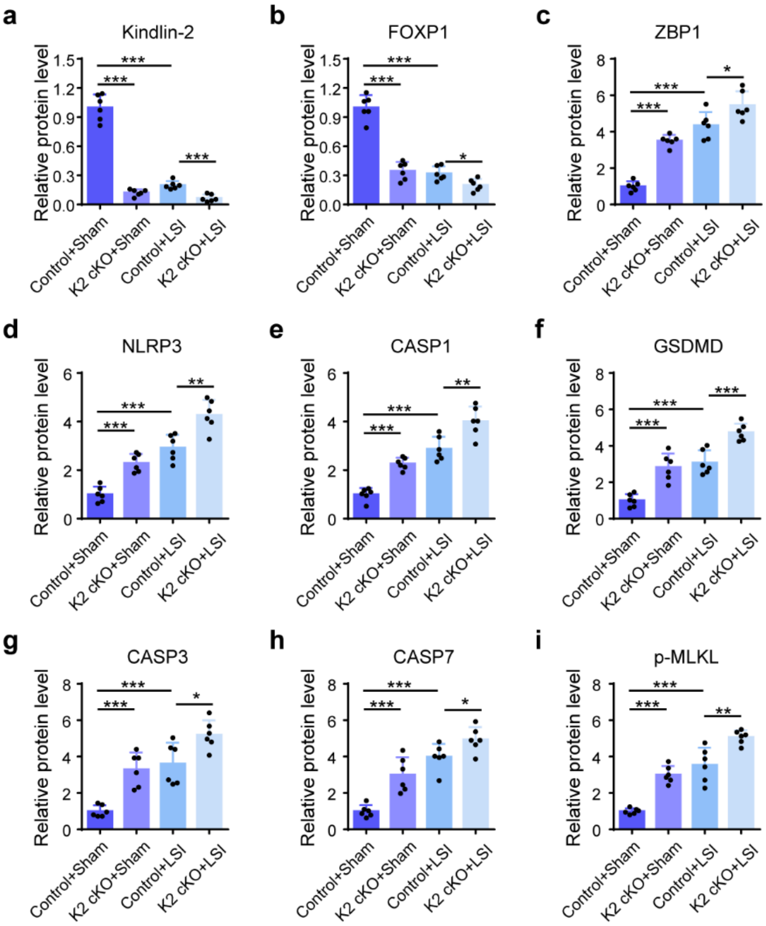

Supplementary Figure 2. **Kindlin-2 deletion promotes NP cell PANoptosis and IDD in the presence of mechanical overloading in mice. (a-i)** IF staining of Kindlin-2, FOXP1, ZBP1, NLRP3, CASP1, GSDMD, CASP3, CASP7, and p-MLKL in NP in control and Kindlin-2 cKO mice with or without lumbar spine instability (LSI). (n=6). Results are expressed as mean ± standard deviation (s.d.). *$P < 0.05$, **$P < 0.01$, ***$P < 0.001$.

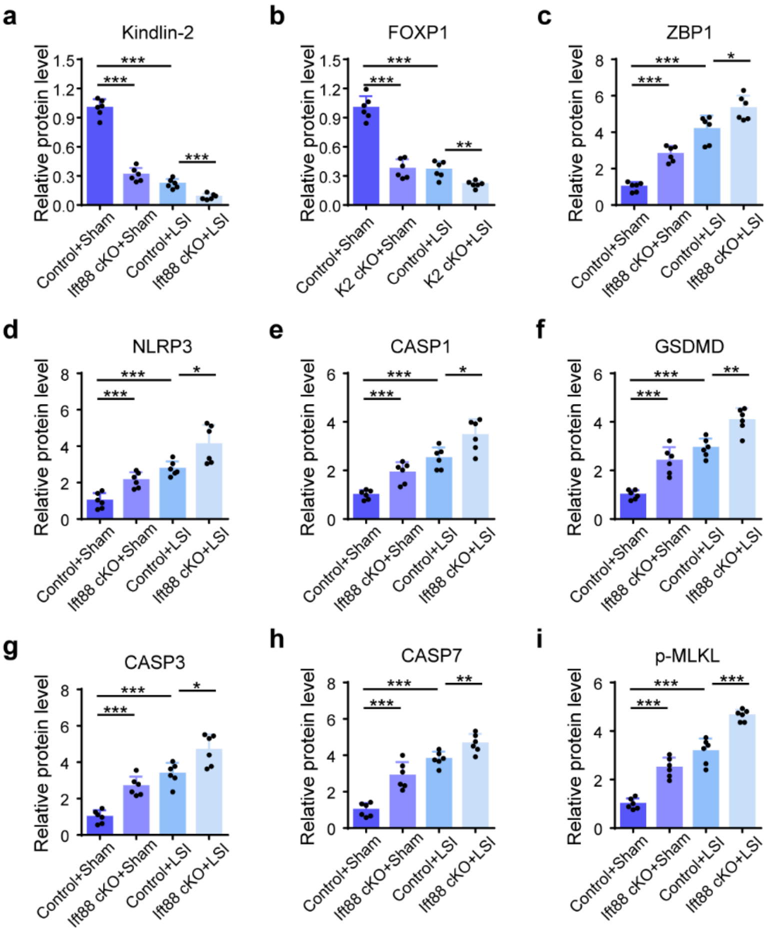

Supplementary Figure 3. **IFT88 deletion decreases Kindlin-2 expression, promotes NP cell PANoptosis and IDD in the presence of mechanical overloading in mice. (a-i)** IF staining of Kindlin-2, FOXP1, ZBP1, NLRP3, CASP1, GSDMD, CASP3, CASP7, and p-MLKL in NP in control and Ift88 cKO mice with or without lumbar spine instability (LSI). (n=6). Results are expressed as mean ± standard deviation (s.d.). **P* < 0.05, ***P* < 0.01, ****P* < 0.001.

Supplementary Tables

Supplementary Table 1. Characteristics details of the patients enrolled in the study.

Case no.	Age (years)	Gender	Diagnosis	Disc level	Pfirrmann grading
Case 1	53	F	Lumbar disc herniation	L5/S1	II
Case 2	49	F	Lumbar disc herniation	L5/S1	II
Case 3	51	M	Lumbar disc herniation	L4/5	III
Case 4	38	M	Lumbar disc herniation	L4/5	III
Case 5	40	F	Lumbar disc herniation	L5/S1	III
Case 6	73	M	Lumbar disc herniation	L4/5	III
Case 7	62	F	Lumbar disc herniation	L5/S1	III
Case 8	59	F	Lumbar disc herniation	L4/5	III
Case 9	33	M	Lumbar disc herniation	L5/S1	IV
Case 10	68	M	Lumbar disc herniation	L5/S1	IV
Case 11	49	F	Lumbar disc herniation	L4/5	IV
Case 12	51	M	Lumbar disc herniation	L4/5	IV
Case 13	60	M	Lumbar disc herniation	L5/S1	V
Case 14	72	F	Lumbar disc herniation	L5/S1	V
Case 15	64	F	Lumbar disc herniation	L4/5	V
Case 16	61	F	Lumbar disc herniation	L5/S1	V

Supplementary Table 2. Primer sequences used in genotyping and sequences used in transfection.

Gene	Forward (5'-3')	Reverse (5'-3')
Kindlin-2	TGTGTTTCAAAGGTACTGGTCA	ACAATGGTGCTTTGCCTACA
Ift88	TTTTCCCGCTGACCTTGTGT	CCAGAGCATGCTACAAGTCAC
Cre	GATCTCCGGTATTGAAACTCCAGC	GCTAAACATGCTTCATCGTCGG

siRNA	Sense (5'-3')	Antisense (5'-3')
Kindlin-2	GCCUCAAGCUCUUCUUGAUTT	AUCAAGAAGAGCUUGAGGCTT
ZBP1-1	GGACAUAGAAAGCUCUCAATT	UUGAGAGCUUUCUAUGUCCTT
ZBP1-2	GCUCCAACAAGUGCAGCUUTT	AAGCUGCACUUGUUGGAGCTT
IFT88	GGACUUAACCUACUCCGUUTT	AACGGAGUAGGUUAAGUCCTT

Supplementary Table 3. Antibody information.

Antibody	Company	Catalog #	Application/Dilution
IFT88	Proteintech	13967-1-AP	WB (1:500); IF (1:200)
Kindlin-2	Proteintech	11453-1-AP	WB (1:1000); IF (1:100)
FOXP1	Proteintech	22051-1-AP	WB (1:1000); IF (1:250)
FOXP1	Abcam	ab314488	WB (1:1000); IF (1:250); IP(1:30)
FLAG	Proteintech	66008-4-Ig	WB (1:10000)
SUMO-1	Abcam	Ab133352	WB (1:1000)
ZBP1	Santa Cruz	sc-271483	WB (1:1000); IF (1:100)
NLRP3	BOSTER	BA3677	WB (1:1000); IF (1:200)
CASP1	ABclonal	A0964	WB (1:1000); IF (1:200)
GSDMD	Proteintech	20770-1-AP	WB (1:500); IF (1:200)
CASP3	ABclonal	A2156	WB (1:1000); IF (1:200)
CASP7	ABclonal	A1524	WB (1:1000); IF (1:200)
p-MLKL	Affbiotech	AF7420	WB (1:500); IF (1:100)
GAPDH	Proteintech	60004-1-Ig	WB (1:3000)
